# Characteristics of children with congenital heart defects and their caregivers in three U.S. sites: the Congenital Heart Survey To Recognize Outcomes Needs and well-beinG of KIDS (CHSTRONG KIDS)^☆^

**DOI:** 10.1016/j.ppedcard.2026.101956

**Published:** 2026-08

**Authors:** Karrie F. Downing, Shannon E. Moss, Sook Ja Cho, Martha M. Werler, Heather Pint, Eric Rubenstein, Vanessa I. Avemil, Ruby Barnard-Mayers, Julianna F. Dick, Karmen Dippmann, Barbara K. Frohnert, Rebecca F. Liberman, Alexis A. Miket, Eirini Nestoridi, Matthew E. Oster, Rebecca Thom, Marissa Tuler, Mahsa M. Yazdy, Yuan Zhang, Emily O. Olsen

**Affiliations:** a National Center on Birth Defects and Developmental Disabilities, Centers for Disease Control and Prevention, Atlanta, GA, United States of America; b Child and Family Health Division, Minnesota Department of Health, St. Paul, MN, United States of America; c Boston University School of Public Health, Boston, MA, United States of America; d Massachusetts Department of Public Health, Boston, MA, United States of America; e Children’s Healthcare of Atlanta, Emory University School of Medicine, Atlanta, GA, United States of America

**Keywords:** Congenital heart defects, Children, Adolescents, Survey, United States, Cardiology

## Abstract

**Background:**

Many US children have congenital heart defects (CHD), yet data on their long-term outcomes have limitations. The Congenital Heart Survey To Recognize Outcomes, Needs, and well-beinG of KIDS (CHSTRONG KIDS) addresses these gaps by surveying caregivers of children with CHD identified through birth defect surveillance systems [BDSS].

**Objectives:**

To describe the CHSTRONG KIDS project design, characteristics of the eligible population, and the percentage not up-to-date on recommended cardiology care.

**Methods:**

Children born 2006–2021 with CHD were identified using active, population-based BDSS in Atlanta, Georgia, Massachusetts, and Minnesota and linked to vital records. Caregivers of eligible (living) children with CHD were invited to complete surveys in 2024–2025. Characteristics were compared by site and response status using χ^2^ tests. We also estimated percentages of children in CHSTRONG KIDS who had not seen a cardiologist within the guideline-recommended timeframe for their specific defects.

**Results:**

Among 7239 identified children with CHD, 6240 were eligible for survey recruitment. Of those,1841 (30%) had caregiver-reported survey data. Several characteristics, including CHD severity, birth year, and Trisomy 21 diagnosis, varied by site (*p* < 0.05). Survey response rates differed by site, CHD severity, maternal race, maternal education, and rurality (*p* < 0.05), prompting development of post-stratification weights. A weighted 21.6% were not up-to-date on their cardiology care.

**Conclusion:**

With data on >7200 CHD cases and >1800 caregiver-reported surveys, CHSTRONG KIDS provides a population-based view of long-term outcomes among children with CHD. Notably, one in five were not up-to-date on their cardiology care and therefore may not be represented in clinical cohorts.

Congenital heart defects (CHD) are the most common type of structural birth defects in the United States, affecting approximately 1 in 110 live-born children [1]. They are a leading cause of birth defect-associated infant mortality, morbidity, and healthcare costs [2–4].

CHD includes a diverse group of cardiac diagnoses of varying phenotypes and severity, each with a specific set of guidelines for care, management, and cardiology follow-up [5]. Given advances in survival, researchers estimate that there are at least 1 million children with CHD in the United States [6].

Given the vast improvements in CHD survival over the past 30 years [2], it is important to evaluate health, social, educational, quality of life, and other outcomes beyond infancy that are specific to those living with CHD in the U.S. Clinical and administrative samples have been used to address long-term outcomes among patient populations with CHD in the U.S. [7–24], but these data usually are not population-based, lack patient-reported outcomes, and only include children who are routinely monitored by a cardiologist. A few population-based national surveys (e.g., National Survey of Children’s Health, National Health Interview Survey) have provided information on health care utilization and other outcomes among children with heart conditions in the U.S. [25–30]. However, these data sources lack detailed and reliable information on the specific type of heart condition, whether the heart condition was congenital or acquired, and other pertinent details at birth (e.g., preterm birth). The dearth of U.S. data on social, educational, quality of life, and other outcomes among children with CHD and their families leaves many public health questions unanswered.

In 2022, the Congenital Heart Survey To Recognize Outcomes, Needs, and well-beinG of KIDS (CHSTRONG KIDS) was launched to characterize long-term outcomes in children born with a select set of CHD. Conducted in three states, CHSTRONG KIDS identified children with CHD from population-based birth defect surveillance data. This project then collected cross-sectional survey data from caregivers on the child’s cardiac and other healthcare utilization, barriers to health care, quality of life, social and educational outcomes, transition of care from childhood to adulthood, survival, other long-term outcomes, and the caregiver’s needs and experiences. CHSTRONG KIDS was funded by the Centers for Disease Control and Prevention (CDC) and modeled after the CH STRONG survey of adults aged 19 to 36 years old with CHD [31]. In this article, we describe features of the CHSTRONG KIDS project design and characteristics of the eligible population, including what percentage are not up-to-date on their cardiology care and therefore may not be represented in clinical or administrative samples.

## 1. Methods

### 1.1. Case identification

Children with select types of CHD born alive between January 1, 2006, and December 31, 2021 (hereafter referred to as “cases”) were identified using population-based birth defect surveillance systems (BDSS) in Massachusetts (MAS), Minnesota (MIN), and metropolitan Atlanta, Georgia (ATL). Each site used active case-finding methods that included review of medical records and classification by an expert clinician. Birth defects were coded using the 6-digit CDC-modified version of the British Paediatric Association (CDC/BPA) coding system, which provides more specificity for birth defects than *the International Classification of Diseases, Ninth and Tenth Revisions, Clinical Modification (ICD-9-CM, ICD-10-CM)*. Sites were only required to identify cases with a subset of CHD codes to be eligible to participate in CHSTRONG KIDS (Appendix A). All sites opted to expand their case inclusion to additional CHD CDC/BPA codes between 745.000 and 747.999 beyond the required list from Appendix A, though not uniformly; therefore, Appendix B provides the list of CHD codes used in CHSTRONG KIDS by site. A brief description of each surveillance system is provided below.

From 1968 to 2011, the Metropolitan Atlanta Congenital Defects Program (MACDP) captured information on children whose major birth defect was diagnosed before the child’s sixth birthday and whose mother resided in the metropolitan Atlanta counties of: Clayton, Cobb, DeKalb, Fulton, or Gwinnett [32]. In 2012, the included counties were reduced to DeKalb, Fulton, and Gwinnett. For all years, trained abstractors visit hospitals across the included counties to find information on children that meet the MACDP definition for having a birth defect. Clinicians with relevant expertise (e.g., pediatric cardiologists) then review the abstracted information for accuracy. Abstracted information and birth certificate data are added to the child’s record. More information about MACDP is located at https://www.cdc.gov/birth-defects/tracking/.

Since 1999, the Massachusetts Birth Defects Monitoring Program (MBDMP) within the Massachusetts Center for Birth Defects Research and Prevention has conducted statewide, population-based active surveillance of birth defects among Massachusetts residents [33,34]. Children with potential birth defects are identified through reports from delivery and specialty hospitals, prenatal diagnostic centers, vital records, and other sources. Maternal, prenatal, and infant records are reviewed by trained medical abstractors to confirm the presence of birth defects identified within 1 year of delivery. All children with a CHD are reviewed by a clinical geneticist trained in pediatric cardiology. Identified children are matched to vital records whenever possible, including searching death files for infant deaths. More information about MBDMP is located at https://www.mass.gov/info-details/about-the-birth-defects-monitoring-program.

The Minnesota Birth Defects Information System (BDIS) was established in mid-2005 and modeled after programs such as MACDP. Population-based surveillance includes children born in Minnesota to Minnesota-resident mothers and diagnosed with selected major structural or genetic anomalies before the first year of age. For 2006–2012 births, surveillance of CHD covered Anoka, Carver, Dakota, Hennepin, Ramsey, Scott, and Washington counties, and expanded to statewide by 2013. Children with potential birth defects are identified through reports from birthing or pediatric hospitals in Minnesota. Additional sources include affiliated clinics reporting through health systems, vital records, and state newborn screening programs. Trained abstractors collect information from medical records and assign preliminary CDC/BPA codes when the birth defect definition was met. Nurses review the abstracts and confirm the codes for each child and, as needed, incorporate a second opinion from a contracted pediatric cardiologist to determine the final CHD diagnosis. All children with CHD for the CHSTRONG KIDS Minnesota site were ascertained from BDIS. More about BDIS is located at https://www.health.state.mn.us/people/childrenyouth/birthdefects/bdis.html.

### 1.2. Birth defect surveillance system data

All identified cases (with CHD) were assigned a unique project ID number. Data elements provided by each BDSS for each case included: child’s birth year, vital status, sex, CHD CDC/BPA codes, and codes for select comorbid congenital anomalies (including CDC/BPA codes 758.000–758.090 for Trisomy 21 used in this analysis), gestational age at birth, birth plurality, birth weight, timing of CHD diagnosis (pre- or postnatal). Additionally, the BDSS provided information about the birth mother (e.g., nationality - born in or outside of the U.S.; race and ethnicity; preferred language - not available for ATL cases; marital status; and educational attainment) and the birth father (race and ethnicity, educational attainment). Cases were categorized into one of five hierarchical CHD severity groups: severe, shunt+valve, shunt only, valve only, or other CHD severity (Appendix B). This categorization is based on the anatomical CHD diagnoses using a similar algorithm as that described in CH STRONG for CDC/BPA [31] and originally in other CHD surveillance programs using ICD-9-CM [35]. Additionally, their primary heart defect was identified as the most severe of their CHDs. If a child had multiple CHDs of similar severity, a CHSTRONG KIDS congenital cardiologist reviewed the diagnosis codes to determine the primary CHD diagnosis. If the cardiologist determined that the individual had >1 primary CHD diagnosis within their most severe category, then the primary diagnosis was categorized as “multiple severe”, “multiple shunt+valve”, “multiple shunt”, “multiple valve”, or “multiple other” accordingly.

### 1.3. Vital records linkage

Vital status for each case was evaluated by sites upon searching state vital records data through August 31, 2022 (for ATL cases) or December 31, 2022 (for MAS and MIN cases) for death certificates that matched the available identifiers (e.g., names, dates of birth, addresses, sex, parent names and dates of birth) in the BDSS. Year of death and underlying cause of death were added to the CHSTRONG KIDS dataset if a match was made with a death certificate. Some additional cases were determined to be deceased through subsequent steps of recruitment and data collection; sites performed updated vital record searches to collect year and cause of death if available. All remaining cases were presumed to be alive.

### 1.4. Tracking and tracing for current contact information

For cases presumed alive and therefore eligible for CHSTRONG KIDS, available identifiers on the caregivers in the BDSS were submitted to tracking and tracing systems (e.g., LexisNexis Accurint, CLEAR) to determine current contact information. Vital status and eligibility for recruitment were updated if a case was reported to be deceased via the tracking and tracing system.

### 1.5. Survey recruitment

Recruitment occurred in phases from January 2024 to April 2025. For eligible cases, sites sent an initial letter to a caregiver’s current address to test the address and to provide information about CHSTRONG KIDS and future recruitment mailings. For MIN and ATL, the initial letter was in English only but included a brief message in Spanish and Somali about how to request the subsequent recruitment materials in either of those languages; Somali was offered because it is known to be nearly as common as Spanish among parents of children with CHD in Minnesota. MAS sent an initial letter in English or Spanish according to the preferred language documented on the birth certificate. This initial mailing included a postage-paid pre-addressed postcard used to decline study participation; all recruitment efforts then ended. Three to four weeks later, sites sent survey packets including: documents explaining the project in plain language, a paper survey, a gift card for a national retailer to be kept regardless of participation, a postage-paid pre-addressed envelope to return the survey, and a QR code/hyperlink for taking the online version of the survey in Research Electronic Data Capture (REDCap) [36,37]. Survey packets were only sent in Spanish or Somali if these were documented as the preferred language in the state surveillance program or if the caregiver requested these translated materials during recruitment; otherwise, the materials were sent in English. REDCap’s Multi-Language Management feature did not include an option for a Somali translation, so the online version of the survey was only offered in English and Spanish. A week later, the MIN and ATL sites mailed a reminder postcard. MAS site staff waited three weeks and then conducted reminder phone calls, during which they offered caregivers the option to complete the survey over the phone. If no response was received within four to five weeks after the first packet mailing, sites mailed a second survey packet with the same documentation as the first, except the gift card, followed by another reminder postcard or phone call.

All mailings directed recipients to the project website www.cdc.gov/chstrongkids for more information. If mailings were returned as undeliverable, the sites re-attempted tracking and tracing to find updated contact information. Respondents who returned a survey were sent a final thank you letter and another gift card. If at any point during recruitment or via the survey, the site was notified that the case was deceased, the case’s vital status was updated, and all recruitment efforts ended.

### 1.6. Survey data collection and entry

The CHSTRONG KIDS survey (Appendix C) included 87 questions for the caregiver on the child’s demographics, heart and general health, healthcare needs, health and cardiac care utilization and experiences, social and educational outcomes, transition to adult care, and barriers to care, as well as the caregiver’s needs, perceptions, and experiences while raising a child with CHD. The majority (*n* = 63) closely mirrored questions from the National Survey of Children’s Health (NSCH; https://www.census.gov/programs-surveys/nsch.html) to allow for comparison between children diagnosed with CHD in CHSTRONG KIDS and children in the general U.S. population or with other caregiver-reported health conditions.

CDC received all completed surveys. Each paper survey was entered into a REDCap database by two independent data entry staff members blinded to the other’s entry. A third person compared the entries and reconciled differences.

BDSS data were de-identified by sites and then sent to CDC to compile. Survey data were linked to the rest of the CHSTRONG KIDS combined dataset (i.e., BDSS, vital status data, project disposition indicators) using the assigned unique project identifiers. Additionally, sites geocoded the birth addresses (from the BDSS) and current addresses at survey completion (from the tracing databases) to determine the respective counties and census tracts of residence. Geography-level socioeconomic and rurality measures were then linked to each case by their geocoded information using the geography level, data source, and data years as described in Appendix D. [38–43] For this analysis, rurality of birth residence was evaluated using the 2013 Rural-Urban Continuum Codes, collapsed into metro or non-metro. The final dataset contains no data linking survey responses to names, addresses, or other identifying information.

### 1.7. Ethics approval

CHSTRONG KIDS was reviewed and approved by the Institutional Review Boards (IRB) at Boston University on April 6, 2023, and the Minnesota Department of Health on February 8, 2023. CDC determined CHSTRONG KIDS to be non-research surveillance on January 25, 2023, and therefore exempt from CDC IRB review. The US Office of Management and Budget approved CHSTRONG KIDS data collection activities (OMB number: 0920-22CL). All survey packets included a participant information sheet describing the purpose and procedures of the project, including the risks, benefits, information safeguards, and voluntary nature of the project. Submission of a completed survey to CDC was taken as consent to participate.

### 1.8. Analysis

For this analysis, we examined the total number of CHD cases ascertained from all BDSS, the number presumed alive and therefore eligible, the number assumed to have accurate and current contact information, and the number for whom a parent or caregiver completed a survey. We described and compared demographic and clinical characteristics at birth for cases across the three sites using χ^2^ tests of association. Using χ^2^ tests, we also compared characteristics at birth of those who completed the survey and those who did not complete the survey for any reason to understand differences between survey respondents and non-respondents. We also provided characteristics at birth by survey response status for each site. *P*-values <0.05 were considered statistically significant. Table cells describing non-missing information for fewer than 10 observations were suppressed. As supplemental analyses, we limited the eligible sample to cases whose defects were included by all three sites (i.e., truncus arteriosus, transposition of great vessels, tetralogy of Fallot, pentalogy of Fallot, pulmonary valve atresia with right ventricle hypoplasia, tricuspid atresia and stenosis, hypoplastic left heart syndrome, coarctation of the aorta, pulmonary artery atresia with ventricular septal defect, and total anomalous pulmonary venous return – all categorized as severe; Appendix B) and re-calculated the distribution of characteristics by site and response status among these cases.

Because characteristics of survey respondents may differ from characteristics of the full eligible population, post-stratification weights were calculated for each survey using site, child sex, maternal race and ethnicity, child age, and CHD severity (all derived from the BDSS data) to address non-response bias. The data elements used to generate post-stratification weights cannot be missing, so we employed random hot deck imputation to populate the missing sex data (missing *n* = 2) and deterministic hot deck nearest neighbor imputation to populate the missing maternal race data (missing *n* = 194). Weights were calculated separately for each site using R version 4.4.0 and the e.calibrate function within the ReGenesees package [44].

Among CHSTRONG KIDS respondents, we provided the number and weighted percentages by primary heart defect. To determine what percentage of the CHSTRONG KIDS population might not be captured in clinical or administrative cohorts, we further calculated weighted percentages of cases who had not seen a cardiologist within the recommended timeframe for their defect or, in other words, those who were not up-to-date on their cardiology care. On the survey, caregivers were asked “When was the last time this child saw a heart doctor?” with the following response options: Less than 1 year, 1–2 years, 3–5 years, more than 5 years, or never saw one. We compared the response to the recommended defect-specific lengths of cardiology follow-up documented in the 2018 American Heart Association and American College of Cardiology Guidelines for the Management of Adults With Congenital Heart Disease [5], similar to CH STRONG analyses [45]. Adult guidelines were used because, to our knowledge, there are no comprehensive U.S. guidelines on follow-up timelines for children with CHD. All heart defects were mapped to their follow-up recommendations under the New York Heart Association classification A, which represents the most lenient follow-up timeframes (Appendix E); for children with multiple heart defects, the shortest specified timeframe was selected. Cases whose last cardiology visit exceeded the recommended timeframe were categorized as not having up-to-date cardiology care. Estimates were calculated overall and stratified by site and CHD severity. Children were excluded from this specific analysis if none of their defects had a follow-up recommendation (i.e., the defects in Appendix E with NYHA Class A Follow-Up documented as “None”) or if their caregiver did not report the last time they saw a cardiologist. As supplemental analyses, we restricted the sample to cases whose defects were included by all three sites and re-calculated the percentage among these cases who were not up-to-date on their cardiology care.

## 2. Results

**Fig. 1.**
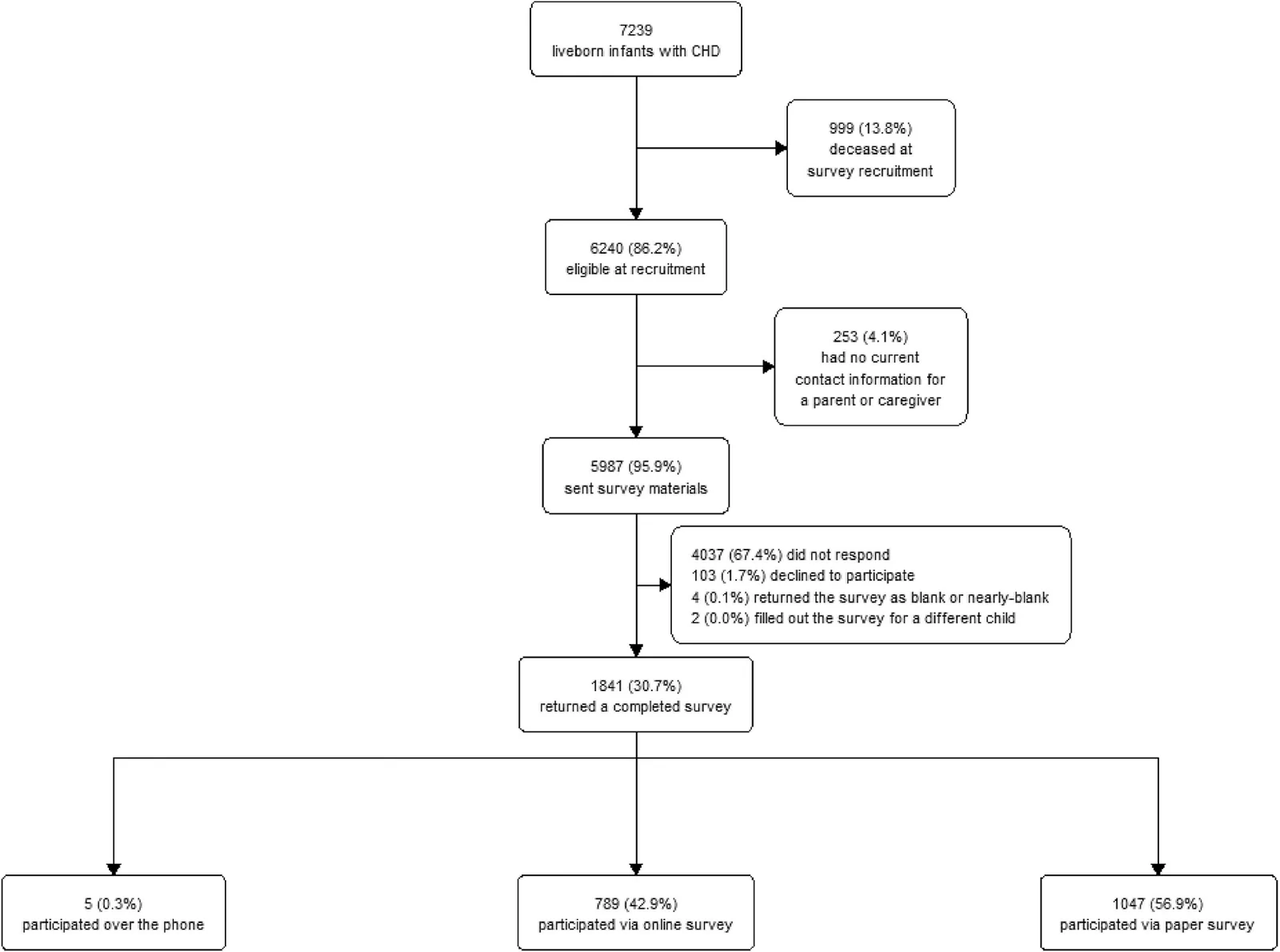
Identification and recruitment of participants for CHSTRONG KIDS. CHD: Congenital heart defects, CHSTRONG KIDS: Congenital Heart Survey To Recognize Outcomes, Needs, and well-beinG of KIDS.

A total of 7239 children born between 2006 and 2021 were identified from the three BDSS with one or more of the CHD codes described in Appendix B (Fig. 1). Of those, 999 (13.8%) were identified as deceased during vital record linkage, tracking and tracing, or survey recruitment, leaving 6240 eligible to participate. Of those, 253 (4.1%) had no valid contact information for a parent or caregiver, and the remaining 5987 were sent survey materials. From January 2024 to April 2025 (when eligible children were between 2 and 19 years old), 1841 parents or caregivers returned a completed survey; 5 (0.3%) over the phone, 789 (42.9%) online, and 1047 (56.9%) via paper survey. Therefore, the survey response rate was 30.7% (using the 5987 for whom recruitment was attempted as the denominator), and the overall response rate was 29.5% (using the 6240 who were eligible to participate as the denominator). Supplemental Fig. 1 includes information on identification and recruitment of respondents further stratified by site. Of identified cases, 2911 (40.2%) were from the ATL BDSS, 1600 (22.1%) were from the MAS BDSS, and 2728 (37.7%) were from the MIN BDSS. Of those, 2591 cases from ATL (89.0%), 1357 cases from MAS (84.8%), and 2292 cases from MIN (84.0%) were presumed alive and eligible to participate. Among those eligible and with valid contact information, 491 caregivers of ATL cases participated (survey response rate = 20.2%, overall response rate = 19.0%), 510 caregivers of MAS participated (survey response rate = 39.0%, overall response rate = 37.6%), and 840 caregivers of MIN participated (survey response rate = 37.3%, overall response rate = 36.6%).

Among eligible cases (Table 1), about half (52.5%) were male, 94.5% were born as singletons, and 59.8% were categorized as having severe CHD. The distribution of several demographic and clinical characteristics differed across the sites (all *p* < 0.01). Specifically, the percentage of male cases ranged from 49.3% (ATL) – 59.4% (MAS), year of birth varied across all strata (e.g., 36.7% [MAS] – 66.7% [ATL] had the earliest birth years, between 2006 and 2011), the percentage born preterm ranged from 14.9% [MIN] – 22.2% [MAS], and the percentage born low birth weight ranged from 16.1% (MAS) – 20.8% (ATL). For CHD severity, only the severe CHD stratum was represented across all sites given the differences in sites’ case inclusion criteria (Appendix B); the proportion of cases with severe CHD therefore ranged from 36.2% (ATL) – 100% (MAS). Across all sites, the percentage of children diagnosed with Trisomy 21 ranged from 4.4% (MAS) – 10.3% (MIN). The percentage of mothers married at delivery ranged from 59.4% (ATL) – 69.5% (MIN). Though there were differences in the distribution of mother’s age and education at delivery, more mothers were between 25 and 34 years old (range: 52.8% [ATL] – 62.1% [MIN]) and had a bachelor’s degree (range: 26.6% [MAS] – 27.7% [ATL]) than the other respective categories. Mother’s race and ethnicity also varied, such that the NH white percentage ranged from 35.1% (ATL) – 71.9% (MIN). Though most cases (94.8%) were born in metro areas, rurality ranged across sites from 0% (ATL) to 13.6% (MIN) non-metro. When limiting to cases with the severe heart defects collected by all three sites (Supplemental Table 1), findings were similar, but the distributions of sex, gestational age, and birth weight no longer significantly differed.

Among cases for whom a survey was received (i.e., respondents; Table 2), participation increased by the child’s year of birth: 24.8% for 2006–2011, 30.9% for 2012 to 2017, and 37.5% for births in 2018–2021. By CHD severity, the highest participation was among caregivers of children with severe CHD (34.7%) and lowest for those with shunt or other CHD (16.6% and 16.3%, respectively). Participation was also higher among caregivers of children with Trisomy 21 (34.1% vs. 29.1%) and increased with maternal age at delivery (12.8% for those ≤18 years – 34.4% for those ≥35 years) and educational attainment (11.3% for those with no HS diploma – 49.5% for those with a graduate degree). Among participants, maternal race was more commonly NH White (40.8%) than any other race. When comparing survey respondents to non-respondents among the eligible population, the distribution of several demographic and clinical variables (site, birth year, gestational age, birth weight, CHD severity, presence of Trisomy 21, maternal marital status, maternal age, educational attainment, maternal race, and rurality) differed (all *p* < 0.05). Supplemental Table 2 presents characteristics of respondents and non-respondents for each site separately. When limiting to cases with the severe heart defects collected by all three sites (Supplemental Table 3), participation was only impacted by maternal sociodemographics at delivery (i.e., marital status, age, educational attainment, race).

**Fig. 2.**
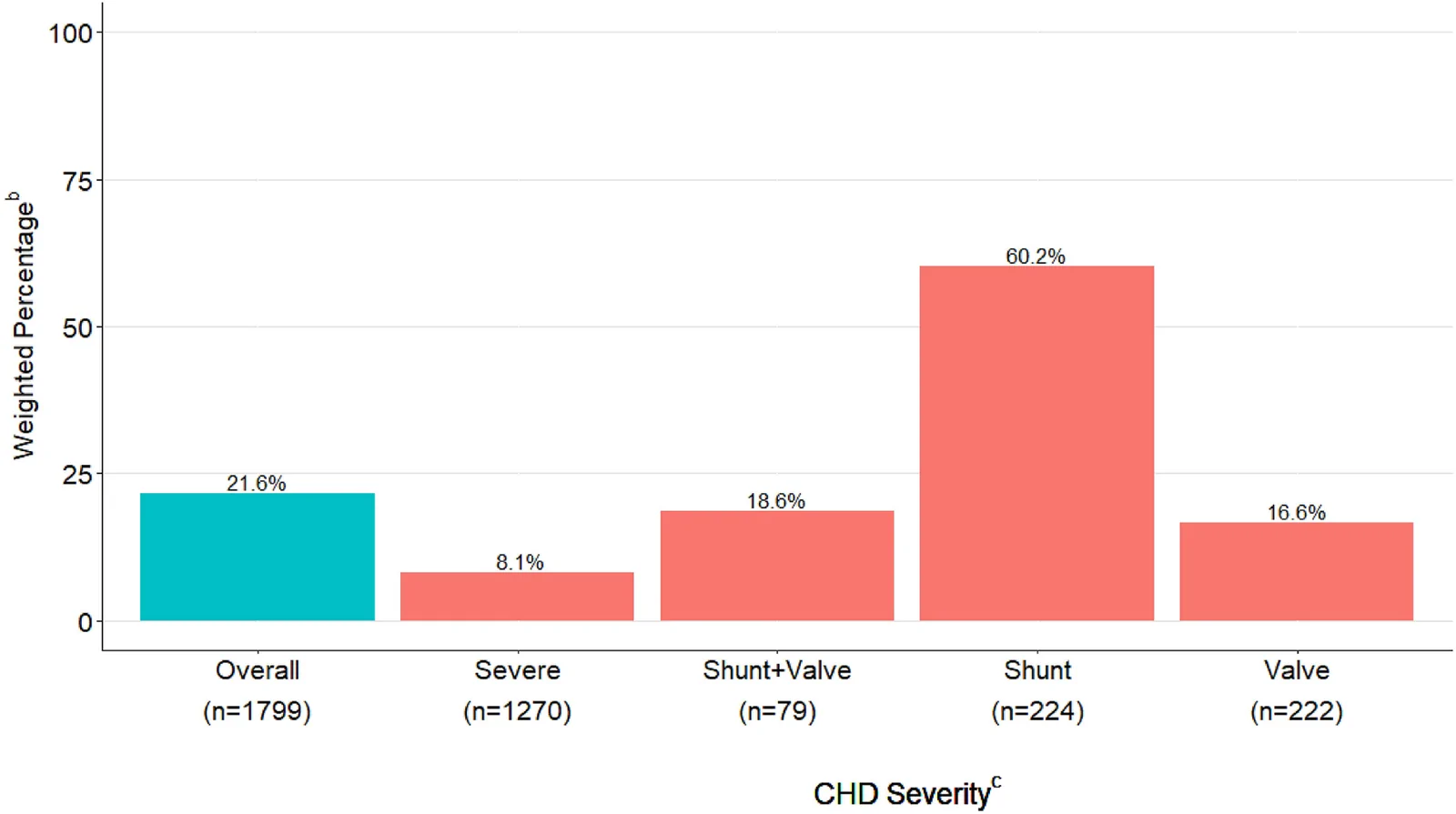
Percentage of children with CHD who were not up-to-date on their cardiology care^a^, CHSTRONG KIDS. CHD: Congenital heart defects, CHSTRONG KIDS: Congenital Heart Survey To Recognize Outcomes, Needs, and well-beinG of KIDS. ^a^ According to recommendations from the 2018 American Heart Association/American College of Cardiology Guideline for the Management of Adults with Congenital Heart Disease [5]. ^b^ Post-stratification weights were calculated for each survey using data on site, child sex, maternal race and ethnicity, child’s age, and CHD severity from the birth defect surveillance systems. ^c^ Estimates for “Other” CHD severity suppressed because *n* < 10.

Table 3 presents the distribution of primary heart defects among the1,841 children of survey respondents. Of these, 31 had children whose heart defects were not described in the 2018 AHA/ACC follow-up recommendations (Appendix E), and 11 did not respond to the survey question “When was the last time this child saw a heart doctor?”; for the remaining 1799 children, we evaluated whether they were up-to-date on their cardiology care (i.e., they saw a cardiologist within the recommended timeframe. A weighted 21.6% of children with CHD overall were not up-to-date on their cardiology care (Fig. 2). About 60.2% of cases with shunt defects were not up-to-date on cardiology care for their specific defects, the highest percentage by CHD severity. However, even among those with severe CHD, 8.1% were not up-to-date. When limiting to cases with heart defects collected at all three sites (all severe) (Supplemental Fig. 2), the overall percentage dropped to 8.1%, and the percentages across sites were more comparable (5.7% [MIN] - 9.8% [MAS]).

## 3. Discussion

To our knowledge, CHSTRONG KIDS is the first population-based US survey of caregivers of children with CHD, designed to document their health, healthcare, social, and life course conditions. We leveraged BDSS data from three U.S. sites, CDC survey methodologies, NSCH questionnaires, and U.S. census data to create a dataset of over 1800 surveys on children with CHDs that is weighted to represent the collective eligible population of 7239 cases across the three sites. Eligible children were born between 2006 and 2021 with specific heart defects that span the milder to the more severe spectrum, resulting in a sample that portrays characteristics across childhood and adolescence. Furthermore, we found that one in five children included in CHSTRONG KIDS were not up-to-date on their cardiology care; therefore, CHSTRONG KIDS data will offer unique insight into the outcomes of children who might not be represented in clinical or administrative cohorts that identify their populations via documentation of recent cardiology care.

We observed some differences in eligible case characteristics across sites (i.e., sex, year of birth, birth weight, CHD severity, maternal sociodemographics, and rurality), likely explained by several factors including diversity of the base populations, differences in the BDSS’ case ascertainment methods, and methods to select cases for inclusion in CHSTRONG KIDS. However, when restricting to the subset of defects that were collected by all three sites, most differences persisted. Even within a site, changes in case ascertainment and inclusion criteria over time could be driving findings; for example, the ATL BDSS’s change in catchment area from 5 to 3 counties in 2012 and the ATL site’s change to a more restrictive defect inclusion over time (Appendix B) are likely responsible for the higher proportion of cases with early birth years.

We observed differences between survey respondents and eligible non-respondents; for example, children of respondents tended to be younger, have more severe CHD, and have mothers with more socioeconomic advantages at delivery (i.e., older, NH white, married, more advanced education). Because the goal of CHSTRONG KIDS is to describe long-term outcomes among all eligible children, the survey data were assigned post-stratification weights to account for differences between respondents and non-respondents, thereby improving generalizability.

Overall, we found that 21.6% of children in CHSTRONG KIDS were not up-to-date on their cardiology care, which is largely driven by cases with non-severe CHD and especially shunt defects (60.2%). When restricting the sample to only the severe defects collected by all three sites, we found a smaller, yet notable, percentage (8.1%) not up-to-date overall and a smaller range across the sites (5.7% [MIN] – 9.8% [MAS]). Because of the unique inclusion criteria for CHSTRONG KIDS and the differences in inclusion criteria by site, the resulting estimates may not be generalizable to all children with CHD. The estimates indicate that the CHSTRONG KIDS dataset includes a substantial proportion of children who have not recently seen a cardiologist and might therefore not be identified without population-based sampling.

The CHSTRONG KIDS program has several predecessors that have evaluated CHD-related long-term outcomes, but these projects have focused on adults rather than children. CHSTRONG KIDS was modeled after CH STRONG, which linked 2016–2019 survey data of young adults with CHD to their BDSS data [31], leading to publications on healthcare access and utilization, survival, comorbidities, socioeconomic factors, and reproductive health. [45,56–63] Other surveys among adults with CHD include the Congenital Heart Disease Project to Understand Lifelong Survivor Experience, the Congenital Heart Initiative: Redefining Outcomes and Navigation to Adult Centered Care, and the Assessment of Patterns of Patient-Reported Outcomes in Adults with Congenital Heart Disease-International Study. [48,50,64,65]

To our knowledge, there are no multi-site, population-based surveys exclusively focusing on long-term outcomes among U.S. children with CHD prior to CHSTRONG KIDS. Researchers have made use of other available data sources to better understand some of the long-term and self- or caregiver-reported outcomes in the pediatric population. NSCH asks caregivers if a doctor or other health care provider ever said that their child had a heart condition. [66–69] Several studies leveraged these data to better understand outcomes among children with CHD, including health and neurodevelopment, healthcare experiences, and adverse childhood experiences, but acknowledge that the presence of a heart condition was not clinically confirmed and can include cardiac conditions that are not CHD. [26,27,70–74] In 2020, the NSCH added a question asking if the child was born with the heart condition; publications using this clarifying question note that it would substantially increase the proportion with CHD, but some of the same limitations remain. [27,73,75,76] The National Health Interview Survey (NHIS) asked caregivers if a doctor or other health professional ever said that their child has congenital heart disease; the question was removed in the 2019 redesign. [77–79] Using these data, researchers have evaluated long-term morbidity of children with CHD and financial hardship among families with CHD. [28,29] Neither the NSCH, nor the NHIS clinically verify the presence of CHD nor do they collect more detailed information on the type of CHD during the surveys. In contrast, this is a strength of CHSTRONG KIDS, since there is access to the BDSS data, the included CHD diagnoses are clinically confirmed and detail each child’s specific diagnoses. Furthermore, the BDSS offers other information at birth from the hospital record, which is useful in determining, for example, the child’s gestational age at birth and comorbid congenital diagnoses like Trisomy 21.

CHSTRONG KIDS has a number of limitations to acknowledge. Across sites, there were differences in the BDSS’ case ascertainment methods and which cases were selected for inclusion. This is particularly important with regard to the proportions of severe versus non-severe types of CHDs, for which many outcomes will vary. It is possible vital status was misclassified; if cases did not link to a death record and were not found deceased prior to recruitment, they were presumed but not confirmed to be alive unless a survey was received. Likewise, if the project received no indication that available contact information was invalid (e.g., no mail returned as undeliverable), cases were assumed but not confirmed to have valid contact information, unless a survey was received; therefore, we may be underestimating the survey response rates by including cases without valid contact information in the denominator. CHSTRONG KIDS does not have clinical data beyond that included in the BDSS; therefore, CHD severity assignment is exclusively based on the child’s native anatomical diagnoses. The survey evaluates several caregiver-reported outcomes that are not clinically or administratively confirmed, e.g., health conditions, schooling, and receipt of healthcare services. However, researchers have demonstrated that parents are able to accurately report their child’s medical care and history. [80–82] The response rate to the CHSTRONG KIDS survey was about 30%, which is lower than preferred but not unusual for a mail-based survey; for comparison, the response rate of the NSCH topical survey in 2023 was about 27%. [83] Response rates differed by site and by child and caregiver characteristics; therefore, post-stratification weights were developed. The estimates from our cardiology care analyses may not be generalizable to children with CHD outside of the CHSTRONG KIDS population because of the unique inclusion criteria specific to each site. A small percentage of children were excluded because the guidelines do not specify a follow-up time period for their CHD or because they were missing data on when they last saw a cardiologist. [5] There are factors to consider that may lead to some under- or over-estimation of cardiology care timeliness. For one, we opted to use the longer period of recommended follow-up when the guidelines offered a range, potentially resulting in underestimation of cardiology care timeliness. Moreover, we used AHA/ACC guidelines that are intended for the management of adults with CHD because there are no comprehensive U. S. pediatric guidelines on follow-up time periods to our knowledge, also potentially resulting in underestimation; cardiologists might prefer to see children with CHD more frequently, e.g., for post-surgical monitoring.

CHSTRONG KIDS has many strengths. Surveys like CHSTRONG KIDS are important because they collect outcomes based on perceptions, knowledge, and experiences that one could only evaluate from self- or caregiver-report. Data collected from CHSTRONG KIDS will describe a variety of long-term outcomes pertaining to the health and well-being of children with CHD and their families. Most outcomes were collected using questions similar to the NSCH to allow for comparison with children without heart conditions and other pediatric populations. CHSTRONG KIDS draws from a large population-based sample of more than 7200 CHD cases identified from active BDSS across three diverse U.S. sites. Among them, over 1800 surveys were collected. Because survey data were linked to data from the BDSS, these data can be used to evaluate outcomes by anatomical severity of the child’s CHD and the specific type of CHD and can also be used to assess associations between characteristics at birth and outcomes at the ages of 2 through 18 years. Furthermore, data from CHSTRONG KIDS will complement research involving clinical or health care data by adding in the caregiver perspective of children who are not regularly being seen by a cardiologist.

## 4. Conclusions

CHSTRONG KIDS is uniquely poised to describe cardiac and other healthcare utilization, barriers to health care, quality of life, social and educational outcomes, transition of care from childhood to adulthood, survival, and other long-term outcomes among a population-based sample of children with CHD, including those who have not recently seen a cardiologist. The survey captures the important perspective of the caregivers, allowing a better understanding of nonclinical outcomes and caregiver experiences that are otherwise challenging to evaluate. Findings from CHSTRONG KIDS will describe how outcomes among children with CHD compare to children without CHD. Furthermore, CHSTRONG KIDS will leverage the detailed diagnoses from the BDSS to describe outcomes by specific CHD type. The information learned from CHSTRONG KIDS may help to inform strategies that improve outcomes for children with CHD and their families.

## Supplementary Material

Supplemental Tables and Figures

Appendices

## Figures and Tables

**Table 1:** Characteristics at birth of children born 2006–2021 with CHD in Atlanta, Georgia, Massachusetts, and Minnesota by site, CHSTRONG KIDS.

Characteristic	Overall n(%) (*N* = 6240)	ATL n(%) (*N* = 2591)	MAS n(%) (*N* = 1357)	MIN n(%) (*N* = 2292)	p-Value^a^
Sex					<0.001
Male	3277 (52.5%)	1277 (49.3%)	806 (59.4%)	1194 (52.1%)	
Female	2961 (47.5%)	1312 (50.7%)	551 (40.6%)	1098 (47.9%)	
Unknown	2	2	0	0	
Year of birth					<0.001
2006–2011	2725 (43.7%)	1729 (66.7%)	498 (36.7%)	498 (21.7%)	
2012–2017	2317 (37.1%)	754 (29.1%)	528 (38.9%)	1035 (45.2%)	
2018–2021	1198 (19.2%)	108 (4.2%)	331 (24.4%)	759 (33.1%)	
Gestational age at birth					<0.001
Preterm (<37 weeks)	1210 (19.5%)	568 (22.2%)	202 (14.9%)	440 (19.2%)	
Full-term (≥ 37 weeks)	4994 (80.5%)	1993 (77.8%)	1150 (85.1%)	1851 (80.8%)	
Unknown	36	30	5	1	
Birth weight (grams)					0.002
<2500 (low)	1196 (19.3%)	536 (20.8%)	214 (16.1%)	446 (19.5%)	
≥2500 (normal)	4998 (80.7%)	2038 (79.2%)	1114 (83.9%)	1846 (80.5%)	
Unknown	46	17	29	0	
Plurality					0.92
Singleton	5861 (94.5%)	2416 (94.5%)	1278 (94.2%)	2167 (94.5%)	
Multiple birth	343 (5.5%)	140 (5.5%)	78 (5.8%)	125 (5.5%)	
Unknown	36	35	1	0	
CHD severity					<0.001
Severe	3734 (59.8%)	939 (36.2%)	1357 (100.0%)	1438 (62.7%)	
Shunt+Valve	288 (4.6%)	60 (2.3%)	NA	228 (9.9%)	
Shunt	1370 (22.0%)	1358 (52.4%)	NA	12 (0.5%)	
Valve	762 (12.2%)	148 (5.7%)	NA	614 (26.8%)	
Other	86 (1.4%)	86 (3.3%)	NA	NA	
Had a cardiac intervention in the first year of life^b^					<0.001
Yes	2287 (81.9%)	NA	951 (70.1%)	1336 (93.1%)	
No	504 (18.1%)	NA	405 (29.9%)	99 (6.9%)	
Unknown	858	NA	1	857	
Diagnosed with Trisomy 21					<0.001
Yes	449 (7.2%)	153 (5.9%)	60 (4.4%)	236 (10.3%)	
No	5791 (92.8%)	2438 (94.1%)	1297 (95.6%)	2056 (89.7%)	
Mother’s marital status at delivery					<0.001
Married	3261 (65.9%)	827 (59.4%)	841 (66.4%)	1593 (69.5%)	
Unmarried	1691 (34.1%)	566 (40.6%)	426 (33.6%)	699 (30.5%)	
Unknown	1288	1198	90	0	
Mother’s age at delivery (years)					<0.001
≤18	148 (2.4%)	87 (3.4%)	29 (2.1%)	32 (1.4%)	
19–24	1037 (16.7%)	515 (20.2%)	198 (14.6%)	324 (14.1%)	
25–34	3530 (57.0%)	1343 (52.8%)	764 (56.3%)	1423 (62.1%)	
≥35	1479 (23.9%)	600 (23.6%)	366 (27.0%)	513 (22.4%)	
Unknown	46	46	0	0	
Mother’s educational attainment at delivery					<0.001
Less than high school	638 (11.2%)	277 (13.1%)	132 (9.9%)	229 (10.1%)	
High school diploma or equivalent	1253 (21.9%)	574 (27.2%)	248 (18.6%)	431 (19.0%)	
Some college, no degree	890 (15.6%)	294 (13.9%)	196 (14.7%)	400 (17.7%)	
Associate degree	488 (8.5%)	105 (5.0%)	139 (10.4%)	244 (10.8%)	
Bachelor’s degree	1552 (27.2%)	584 (27.7%)	355 (26.6%)	613 (27.1%)	
Graduate degree	891 (15.6%)	278 (13.2%)	265 (19.9%)	348 (15.4%)	
Unknown	528	479	22	27	
Mother’s race and ethnicity					<0.001
Non-Hispanic White	3353 (55.4%)	855 (35.1%)	863 (64.7%)	1635 (71.8%)	
Non-Hispanic Black	1334 (22.1%)	919 (37.7%)	124 (9.3%)	291 (12.8%)	
Hispanic	948 (15.7%)	542 (22.2%)	234 (17.6%)	172 (7.6%)	
Non-Hispanic					
Native American or Alaskan Native	25 (0.4%)	<10^c^	<10^c^	24 (1.1%)	
Non-Hispanic Asian or Pacific Islander	358 (5.9%)	115 (4.7%)	95 (7.1%)	148 (6.5%)	
Non-Hispanic Other	30 (0.5%)	<10^c^	17 (1.3%)	<10^c^	
Unknown	192	152	24	16	
Rurality of birth residence					<0.001
Metro	5908 (94.8%)	2587 (100.0%)	1341 (98.9%)	1980 (86.4%)	
Non-metro	327 (5.2%)	0 (0.0%)	15 (1.1%)	312 (13.6%)	
Unknown	5	4	1	0	

ATL: Atlanta site, CHSTRONG KIDS: Congenital Heart Survey To Recognize Outcomes, Needs, and well-beinG of KIDS, NA: Not Available, MAS: Massachusetts site, MIN: Minnesota site.

a Fisher’s Exact test for mother’s race and ethnicity; Pearson’s Chi-squared test for all other comparisons.

b Ns and %s for cardiac intervention exclude ATL cases (for whom data was not available).

c Cells with <10 observations suppressed for known categories.

**Table 2:** Survey response rates by characteristics at birth among 6240 children eligible for CHSTRONG KIDS.

Characteristic	Respondents n(row %) (*N* = 1841)	Non-respondents n(row %) (*N* = 4399)	p-Value^a^
Sex			0.060
Male	1001 (30.5%)	2276 (69.5%)	
Female	840 (28.4%)	2121 (71.6%)	
Unknown	0	2	
Year of birth			<0.001
2006–2011	676 (24.8%)	2049 (75.2%)	
2012–2017	716 (30.9%)	1601 (69.1%)	
2018–2021	449 (37.5%)	749 (62.5%)	
Gestational age at birth			0.022
Preterm (<37 weeks)	325 (26.9%)	885 (73.1%)	
Full-term (≥37 weeks)	1509 (30.2%)	3485 (69.8%)	
Unknown	7	29	
Birth weight (grams)			0.012
<2500 (low)	317 (26.5%)	879 (73.5%)	
≥2500 (normal)	1510 (30.2%)	3488 (69.8%)	
Unknown	14	32	
Plurality			0.77
Singleton	1734 (29.6%)	4127 (70.4%)	
Multiple birth	104 (30.3%)	239 (69.7%)	
Unknown	3	33	
CHD severity			<0.001
Severe	1294 (34.7%)	2440 (65.3%)	
Shunt+Valve	81 (28.1%)	207 (71.9%)	
Shunt	227 (16.6%)	1143 (83.4%)	
Valve	225 (29.5%)	537 (70.5%)	
Other	14 (16.3%)	72 (83.7%)	
Had a cardiac intervention in the first year of life^b^			0.11
Yes	894 (39.1%)	1393 (60.9%)	
No	178 (35.3%)	326 (64.7%)	
Unknown	278	580	
Has Trisomy 21			0.03
Yes	153 (34.1%)	296 (65.9%)	
No	1688 (29.1%)	4103 (70.9%)	
Mother’s marital status at delivery			<0.001
Married	1245 (38.2%)	2016 (61.8%)	
Unmarried	324 (19.2%)	1367 (80.8%)	
Unknown	272	1016	
Mother’s age at delivery			<0.001
≤18	19 (12.8%)	129 (87.2%)	
19–24	185 (17.8%)	852 (82.2%)	
25–34	1120 (31.7%)	2410 (68.3%)	
≥35	509 (34.4%)	970 (65.6%)	
Unknown	8	38	
Mother’s educational attainment at delivery			<0.001
Less than high school	72 (11.3%)	566 (88.7%)	
High school diploma or equivalent	229 (18.3%)	1024 (81.7%)	
Some college, no degree	239 (26.9%)	651 (73.1%)	
Associate degree	162 (33.2%)	326 (66.8%)	
Bachelor’s degree	625 (40.3%)	927 (59.7%)	
Graduate degree	441 (49.5%)	450 (50.5%)	
Unknown	73	455	
Mother’s race and ethnicity			<0.001
Non-Hispanic White	1369 (40.8%)	1984 (59.2%)	
Non-Hispanic Black	193 (14.5%)	1141 (85.5%)	
Hispanic	143 (15.0%)	806 (85.0%)	
Non-Hispanic American Indian and Alaskan Native	<10^c^	22 (88.0%)	
Non-Hispanic Asian or Pacific Islander	99 (27.7%)	259 (72.3%)	
Non-Hispanic other	<10^c^	26 (86.7%)	
Unknown	31	161	
Rurality of birth residence			0.011
Metro	1724 (29.2%)	4184 (70.8%)	
Non-metro	117 (35.8%)	210 (64.2%)	
Unknown	0	5	

ATL: Atlanta site, CHSTRONG KIDS: Congenital Heart Survey To Recognize Outcomes, Needs, and well-beinG of KIDS, MAS: Massachusetts site, MIN: Minnesota site, NA: Not Available.

a Fisher’s Exact test for mother’s race and ethnicity; Pearson’s Chi-squared test for all other comparisons.

b Ns and %s for cardiac intervention exclude ATL cases (for whom data was not available).

c Cells with <10 observations suppressed for known categories.

**Table 3:** Primary heart defect type among 1841 children of CHSTRONG KIDS survey respondents.

Primary heart defect^a^	n	Weighted % (95% CI)^b^
Severe		
Coarctation of the aorta	316	14.1 (12.5–15.7)
Common atrioventricular canal/Endocardial cushion defects	136	6.1 (5.0–7.2)
Corrected transposition of great vessels	15	0.7 (0.3–1.2)
Double outlet right ventricle	40	1.9 (1.2–2.5)
Hypoplastic left heart syndrome	86	4.1 (3.2–5.0)
Multiple severe	47	2.2 (1.5–2.8)
Pulmonary valve atresia/hypoplasia	39	2.0 (1.3–2.7)
Single ventricle	14	0.5 (0.2–0.8)
Tetralogy of Fallot	301	13.9 (12.3–15.5)
Total anomalous pulmonary venous return	48	2.6 (1.8–3.3)
Transposition of the great vessels	170	7.3 (6.1–8.5)
Tricuspid valve anomalies non-atretic	17	1.2 (0.6–1.8)
Tricuspid valve atresia	34	1.7 (1.0–2.3)
Truncus arteriosus	24	1.1 (0.6–1.5)
Shunt +valve		
Multiple shunt + valve	58	2.9 (2.1–3.7)
Valve		
Aortic valve anomalies	41	2.1 (1.4–2.7)
Ebstein’s anomaly	17	0.8 (0.4–1.1)
Pulmonary valve and subpulmonary obstructive anomalies, including atresia	187	10.7 (9.1–12.2)
Shunt		
Atrial septal defect	49	4.9 (3.5–6.3)
Multiple shunt	12	1.4 (0.6–2.1)
Ventricular septal defect	163	15.6 (13.5–17.6)

CHSTRONG KIDS: Congenital Heart Survey To Recognize Outcomes, Needs, and well-beinG of KIDS.

a Types with <10 observations are not shown but include aortic arch and vascular ring anomalies (other severity), cor triatriatum (other severity), coronary artery and sinus anomalies (other severity), interrupted aortic arch (severe), multiple valve (valve), other atrial defects (other severity), other specified aortic anomalies (other severity), and primum defects and common atrium (shunt).

b Post-stratification weights were calculated for each survey using data on site, child sex, maternal race and ethnicity, child’s age and CHD severity from the birth defect surveillance systems.
