# Supplementary material for: Characteristics of children with congenital heart defects and their caregivers in three U.S. sites: the Congenital Heart Survey To Recognize Outcomes Needs and well-beinG of KIDS (CHSTRONG KIDS)
Source: Prog Pediatr Cardiol. Author manuscript; Available in PMC 2026 Sep 19. (PMC13588542; doi:10.1016/j.ppedcard.2026.101956)
Supplement: Supplementary file 1 [file NIHMS2198443-supplement-Appendices.docx]

##### **Appendix A. Minimum diagnosis code list of congenital heart defect cases to be identified by sites to participate in CHSTRONG KIDS**

| **ICD-9-BPA** | **Description** |
| --- | --- |
| 745.000 | Truncus arteriosus |
| 745.100 | Dextro-transposition of great arteries with intact ventricular septum |
| 745.110 | Dextro-transposition of great arteries with ventricular septal defect |
| 745.200 | Tetralogy of Fallot |
| 745.200 plus 746.020 | Tetralogy of Fallot plus absent pulmonary valve |
| 745.200 plus 745.510 | Pentalogy of Fallot |
| 746.000 | Pulmonary valve atresia/intact ventricular septum |
| 746.100 | Tricuspid atresia |
| 746.700 | Hypoplastic left heart syndrome |
| 747.100, 747.110, 747.120, or 747.190 | Coarctation of the aorta |
| 747.310 | Pulmonary atresia with VSD (tetralogy of Fallot variant) |
| 747.420 | Total anomalous pulmonary venous return/connection/drainage |

CHSTRONG KIDS: Congenital Heart Survey To Recognize Outcomes, Needs and well-beinG of KIDS; ICD-9-BPA: International Classification of Diseases, Ninth Revision, British Pediatric Association

##### **Appendix B. All congenital heart defect (CHD)-related Centers for Disease Control and Prevention (CDC)-modified version of the British Pediatric Association (BPA) codes used by sites to identify CHSTRONG KIDS cases from 2006-2021, by CHD severity**

| **Code meets inclusion criteria for surveillance system? :** | | | **ATL** | **MAS** | **MIN** |  |
| --- | --- | --- | --- | --- | --- | --- |
| **Severe Codes** | | | | | | |
| 745.000 | Truncus arteriosus | | Y | Y | Y |  |
| 745.010 | Aortic septal defect / Aortopulmonary window | | Y^1^ |  |  |  |
| *Transposition and Double Outlet Right Ventricle* | | | Y | Y | Y |  |
|  | 745.100 | Transposition of great vessels, complete (no VSD) |  |  |  |  |
|  | 745.110 | Transposition of great vessels, incomplete (w/ VSD), Taussig Bing | Y | Y | Y |  |
|  | 745.120 | Corrected transposition of great vessels, L transposition, vent inversion | Y^1^ |  | Y |  |
|  | 745.130 | Double outlet right ventricle - normally related great vessels | Y^1^ |  | Y |  |
|  | 745.140 | Double outlet right ventricle - transposed great vessels | Y^1^ |  | Y |  |
|  | 745.150 | Double outlet right ventricle - relationship of great vessels NOS | Y^1^ |  | Y |  |
|  | 745.180 | Transposition of great vessels (TGA, TGV) - Other specified | Y^2^ |  | Y |  |
|  | 745.190 | Unspecified transposition of great vessels | Y^1^ |  | Y |  |
| 745.200 | Tetralogy of Fallot | | Y | Y | Y |  |
| 745.210 | Fallot’s pentalogy (tetralogy plus ASD) | | Y^1^ | Y | Y |  |
| *Single ventricle* | | |  |  |  |  |
|  | 745.300 | Single ventricle, not specified | Y^2^ |  | Y |  |
|  | 745.310 | Single ventricle, Double Inlet Left Ventricle | Y^2^ |  | Y^3^ |  |
|  | 745.320 | Single ventricle, Double Inlet Right Ventricle | Y^2^ |  | Y^3^ |  |
|  | 745.330 | Single ventricle, Indeterminate Ventricular Morphology | Y^2^ |  | Y^3^ |  |
|  | 745.380 | Single ventricle, Other Specified | Y^2^ |  | Y^3^ |  |
| 745.620 | Common atrioventricular canal with VSD | | Y^2^ |  | Y |  |
| 745.630 | Common atrioventricular canal | | Y^2^ |  | Y |  |
| 745.680 | Other specified cushion defect | | Y^2^ |  | Y |  |
| 745.690 | Endocardial cushion defect, NOS | | Y^2^ |  | Y |  |
| 746.000 | Pulmonary valve atresia, hypoplasia | | Y | Y | Y |  |
| 746.030 | Pulmonary valve atresia with VSD | | Y | Y |  |  |
| 746.100 | Tricuspid atresia and stenosis | | Y | Y | Y |  |
| 746.106 | Tricuspid valve - Stenosis or hypoplasia, congenital | | Y^1^ |  |  |  |
| 746.505 | Absence, atresia, or hypoplasia of mitral valve | | Y^1^ |  |  |  |
| 746.700 | Hypoplastic Left Heart Syndrome | | Y | Y | Y |  |
| *Coarctation of the aorta (all types)* | | |  |  |  |  |
|  | 747.100 | Pre-ductal | Y | Y | Y |  |
|  | 747.110 | Post-ductal | Y | Y | Y^4^ |  |
|  | 747.120 | Ductal | Y | Y | Y^4^ |  |
|  | 747.190 | Coarctation of the aorta, NOS | Y | Y | Y^4^ |  |
| 747.200 | Aortic atresia (including pseudotruncus arteriosus) | | Y^1^ |  |  |  |
| 747.210 | Aortic hypoplasia | | Y^1^ |  |  |  |
| *Interrupted aortic arch* | | |  |  |  |  |
|  | 747.215 | Interrupted aortic arch - Type A | Y^2^ |  |  |  |
|  | 747.216 | Interrupted aortic arch - Type B | Y^2^ |  |  |  |
|  | 747.217 | Interrupted aortic arch - Type C | Y^2^ |  |  |  |
|  | 747.285 | Interrupted aortic arch, NOS | Y^2^ |  |  |  |
| 747.310 | Pulmonary artery atresia with septal defect | | Y | Y | Y |  |
| 747.420 | Total Anomalous Pulmonary Venous Return | | Y | Y | Y^5^ |  |
| **Shunt + Valve Codes**  *Note: Additional cases will be in this category if they have codes in both the Shunt and Valve categories below.* | | | | | | |
| 745.520 | Lutembacher syndrome | | Y^1^ |  |  |  |
| 746.840 | Trilogy of Fallot | | Y^1^ |  |  |  |
| **Shunt Codes** | | | | | | |
| *Ventricular Septal Defect, (all types)* | | |  |  |  |  |
|  | 745.400 | Roger's disease | Y^1^ |  |  |  |
|  | 745.410 | Eisenmenger's syndrome | Y^1^ |  |  |  |
|  | 745.420 | Gerbode defect | Y^1^ |  |  |  |
|  | 745.480 | Other specified ventricular septal defect | Y^1^ |  |  |  |
|  | 745.485 | Ventricular septal defect - Perimembranous or membranous | Y^2^ |  |  |  |
|  | 745.486 | Ventricular septal defect - Muscular, mid-muscular, apical | Y^2^ |  |  |  |
|  | 745.487 | Ventricular septal defect - Inlet | Y^2^ |  |  |  |
|  | 745.490 | Ventricular septal defect, NOS | Y^2^ |  |  |  |
| *Atrial Septal Defect (all types)* | | | Y^2^ |  |  |  |
|  | 745.510 | Secundum atrial septal defect |  |  |  |  |
|  | 745.580 | Other specified atrial septal defect | Y^2^ |  |  |  |
|  | 745.590 | Atrial septal defect, NOS | Y^2^ |  |  |  |
| 745.600 | Ostium primum defects | | Y^2^ |  | Y |  |
| 745.610 | Single common atrium, cor triloculare biventriculare | | Y^2^ |  | Y |  |
| 745.800 | Other bulbus cordis anomalies and anomalies of cardiac septal closure | | Y^1^ |  |  |  |
| 745.900 | Unspecified defect of septal closure | | Y^1^ |  |  |  |
| 747.430 | Partial anomalous pulmonary venous return | | Y^2^ |  |  |  |
| **Valve Codes** | | | | | | |
| 746.010 | Pulmonary valve stenosis | | Y^2^ |  | Y |  |
| 746.830 | Pulmonary infundibular stenosis | | Y^1^ |  |  |  |
| 746.080 | Other specified anomalies of the pulmonary valve | | Y^1^ |  |  |  |
| 746.090 | Unspecified anomaly of pulmonary valve | | Y^1^ |  |  |  |
| 746.200 | Ebstein anomaly | | Y^2^ |  | Y |  |
| 746.300 | Aortic valve stenosis (includes valvar and subvalvar) | | Y^2^ |  | Y |  |
| 746.470 | Bicuspid aortic valve | | Y^1^ |  |  |  |
| 746.480 | Other specified anomalies of aortic valve (including aortic valve atresia) | | Y^1^ |  |  |  |
| 746.490 | Unspecified anomalies of aortic valve | | Y^1^ |  |  |  |
| 746.500 | Congenital mitral stenosis | | Y^1^ |  |  |  |
| 746.900 | Unspecified anomalies of heart valves | | Y^1^ |  |  |  |
| 746.995 | Pulmonic or pulmonary atresia, stenosis, or hypoplasia, NOS | | Y^1^ |  |  |  |
| 747.220 | Supra-aortic stenosis (supra-valvar) | | Y^1^ |  |  |  |
| 747.300 | Pulmonary artery atresia, absence, or agenesis | | Y^1^ |  |  |  |
| **Other Codes** | | | | | | |
| 745.700 | Cor biloculare | | Y^1^ |  |  |  |
| 746.820 | Cor triatriatum | | Y^1^ |  |  |  |
| 746.881 | Hypoplastic left ventricle | | Y^1^ |  |  |  |
| 746.882 | Hypoplastic right heart/ ventricle (Uhl's disease) | | Y^1^ |  |  |  |
| 746.883 | Hypoplastic ventricle, NOS | | Y^1^ |  |  |  |
| 746.885 | Coronary artery anomaly | | Y^1^ |  |  |  |
| 746.887 | Other defects of the atria | | Y^1^ |  |  |  |
| 746.910 | Anomalous bands of heart | | Y^1^ |  |  |  |
| 746.920 | Acyanotic congenital heart disease, NOS | | Y^1^ |  |  |  |
| 746.930 | Cyanotic congenital heart disease, NOS | | Y^1^ |  |  |  |
| *Anomalies of aorta (all types)* | | |  |  |  |  |
|  | 747.230 | Persistent right aortic arch | Y^1^ |  |  |  |
|  | 747.240 | Aneurysm of sinus of Valsalva | Y^1^ |  |  |  |
|  | 747.250 | Vascular ring (includes double aortic arch) | Y^1^ |  |  |  |
|  | 747.260 | Overriding aorta | Y^1^ |  |  |  |
|  | 747.270 | Aortic aneurysm | Y^1^ |  |  |  |
|  | 747.280 | Other specified anomalies of aorta | Y^1^ |  |  |  |
|  | 747.290 | Unspecified anomalies of aorta | Y^1^ |  |  |  |
| 747.400 | Stenosis of vena cava (inferior or superior) | | Y^1^ |  |  |  |
| 747.410 | Persistent left superior vena cava | | Y^1^ |  |  |  |

ASD: Atrial septal defect; CHSTRONG KIDS: Congenital Heart Survey To Recognize Outcomes, Needs and well-beinG of KIDS; NOS: Not otherwise specified; VSD: Ventricular septal defect; Y: Yes

^1^ Only for birth years between 2006-2008 and 2014-2015

^2^ Only for birth years between 2006-2013

^3^All single ventricle defect subtypes are coded as 745.300 by the MIN surveillance system; codes to distinguish subtypes are not used by the system but all eligible single ventricle cases were identified for CHSTRONG KIDS

^4^ Only for birth years between 2006-2012; for 2013 and later, all coarctation of aorta subtypes are coded as 747.100 by the MIN surveillance system

^5^ Only for birth years between 2013-2021

**Case does not meet inclusion criteria for CHSTRONG KIDS if they have no other CHD codes besides the following:**

745.xx8, 746.xx8, 747.xx8	ANY CHD code which is considered “possible or probable”, including those using an “8” as the 6th digit.

745.500	Patent foramen ovale

746.020	Pulmonary insufficiency

746.105	Tricuspid valve insufficiency

746.400	Aortic insufficiency/Bicuspid aortic valve

746.600	Mitral insufficiency

746.800	Dextrocardia

746.810	Levocardia

746.850	Anomalies of pericardium

746.860	Anomalies of myocardium

746.870	Congenital heart block

746.880	Other specified anomaly of heart

746.886	Ventricular hypertrophy

746.990	Unspecified anomalies of heart

747.000	Patent ductus arteriosus

747.320	Pulmonary artery stenosis

747.325	Peripheral pulmonary artery stenosis

747.330	Aneurysm of pulmonary artery

747.340	Pulmonary arteriovenous malformations

747.380	Other specified anomaly of pulmonary artery

747.390	Unspecified anomaly of pulmonary artery

747.440	Anomalous portal vein termination

747.450	Portal-hepatic vein fistula

747.480	Other specified anomalies of great veins

747.490	Unspecified anomalies of great veins

747.500	Single umbilical artery

747.6xx	Other anomalies of peripheral vascular system, including:

747.600	Stenosis of renal artery

747.610	Other anomalies of renal artery

747.620	Arteriovenous malformation (peripheral)

747.630	Congenital phlebectasia, congenital varix

747.640	Other anomalies of peripheral arteries, including aberrant subclavian artery

747.650	Other anomalies of peripheral veins

747.680	Other anomalies of peripheral vascular system

747.690	Unspecified anomalies of peripheral vascular system

747.8xx	Other specified anomalies of circulatory system, including:

747.800	Arteriovenous (malformation) aneurysm of brain

747.810	Other anomalies of cerebral vessels

747.880	NOS anomalies of circulatory system

747.9xx	Unspecified anomaly of circulatory system

**Appendix C. English survey instrument for the Congenital Heart Survey To Recognize Outcomes, Needs, and well-beinG of KIDS**

**Congenital Heart Survey To Recognize Outcomes, Needs, and well-beinG of KIDS (CHSTRONG KIDS)**

As explained in the letter you received with this survey, we are contacting you about this survey because our records show that your child was born with a heart condition. We would like to ask you some questions about your child, their health, and your family.

The survey should be completed by a parent or adult caregiver who lives in this household and who is familiar with your child’s health and health care.

Please answer questions with information about your child with a heart condition only. **You may choose to skip any question you do not wish to answer.**

**Your child’s information**

What is this child’s age in years?

Is this child Hispanic or Latino?

- Hispanic or Latino
- Not Hispanic or Latino

What is this child’s race? *(Select all that apply)*

American Indian or Alaska Native alone

Asian

Black or African American

Native Hawaiian or Other Pacific Islander

White

**Your child’s heart condition**

What is the name of the heart condition that this child was born with? *(Select all that apply)*

Aortic valve stenosis

Atrial septal defect (ASD)

Atrioventricular septal defect (AVSD) or Atrioventricular canal (AV canal)

Bicuspid aortic valve

Coarctation of aorta

Ebstein anomaly

Hypoplastic left heart syndrome (HLHS)

Patent ductus arteriosus (PDA)

Pulmonary atresia

Pulmonary valve stenosis

Single ventricle (double inlet left ventricle)

Tetralogy of Fallot (TOF)

Transposition of the great arteries (TGA)

Tricuspid atresia

Truncus arteriosus

Ventricular septal defect (VSD)

Other *(Please specify)*

Don’t know/not sure

No heart condition that I know of ***(Please answer remaining questions to the best of your ability)***

Has this child ever had surgery for the heart condition they were born with? Heart surgery will result in scars on the middle of the chest, side, or back.

Yes

No

Don’t know/not sure

What type of information or help should be available to caregivers of children born with a heart condition?

When this child was first diagnosed with a heart condition, were you provided enough information on what this meant for their emotional, social and physical health?

Yes

No

Don’t know/not sure

**Your child’s heart doctors**

When was the last time this child saw a heart doctor?

Less than 1 year

1-2 years

3-5 years

Go to question 13

More than 5 years

Never saw one

How many health care provider visits were with a heart doctor or at a cardiology clinic (clinic that only sees patients with heart conditions) in the past 12 months?

Numeric response:

Who are the majority of patients that this child’s primary heart doctor usually sees?

Children and adolescents (pediatric cardiologist)

Adults (adult congenital heart cardiologist or adult cardiologist)

Go to question 12

Don’t know/not sure

Has a doctor or other health care provider talked with you about when this child will need to see heart doctors who treat adults (adult congenital heart cardiologist or adult cardiologist)?

Yes

No

In the past 2 years, how often has this child’s heart doctor…?

|  | Always | Usually | Sometimes | Never |
| --- | --- | --- | --- | --- |
| a. Spent enough time with this child | ¡ | ¡ | ¡ | ¡ |
| b. Listened carefully to you | ¡ | ¡ | ¡ | ¡ |
| c. Shown sensitivity to your family’s values and customs | ¡ | ¡ | ¡ | ¡ |
| d. Provided the specific information you needed concerning this child | ¡ | ¡ | ¡ | ¡ |
| e. Helped you feel like a partner in this child’s care | ¡ | ¡ | ¡ | ¡ |
| f. Discussed with you the range of options to consider for this child’s health care or treatment | ¡ | ¡ | ¡ | ¡ |
| g. Made it easy for you to raise concerns or disagree with recommendations for this child’s health care | ¡ | ¡ | ¡ | ¡ |
| h. Worked with you to decide together which health care and treatment choices would be best for this child | ¡ | ¡ | ¡ | ¡ |

***Go to instructions before question 14.***

If this child has not seen a heart doctor in the last 2 years or ever, why? *(Select all that apply)*

This child felt well

Did not think this child needed to see a heart doctor

Doctor told me this child no longer needed to see a heart doctor

Changed or lost insurance

Moved to a different city or town

Did not like this child’s heart doctor

Couldn’t find a heart doctor

I had too many other things going on

There were issues related to cost

I chose to postpone or cancel appointments due to COVID-19

This child’s heart doctor postponed or cancelled appointments due to COVID-19

Other *(Please specify)*

**Your child’s general health**

***If your child is younger than 6 years old, go to question 16.***

What is this child’s CURRENT height? *(Answer in either feet and inches or meters and centimeters)*

______ feet AND ______ inches

______ meters AND ______ centimeters

How much does this child CURRENTLY weigh? *(Answer in either pounds or kilograms)*

______ pounds

______ kilograms

In general, how would you describe this child’s health?

Excellent

Very good

Good

Fair

Poor

**Your child’s medical conditions**

Has a doctor or other health care provider EVER told you that this child has…?

|  | Yes | No |
| --- | --- | --- |
| a. Anxiety problems | ¡ | ¡ |
| b. Depression | ¡ | ¡ |
| c. Developmental delay | ¡ | ¡ |
| d. Behavioral or conduct problems | ¡ | ¡ |
| e. Intellectual disability | ¡ | ¡ |
| f. Speech or other language disorder | ¡ | ¡ |
| g. Learning disability | ¡ | ¡ |
| h. Attention Deficit Disorder or Attention Deficit / Hyperactivity Disorder, that is, ADD or ADHD | ¡ | ¡ |
| i. Autism, Autism Spectrum Disorder, Asperger’s Disorder, or Pervasive Developmental Disorder (PDD) | ¡ | ¡ |
| j. Diabetes | ¡ | ¡ |
| k. Down Syndrome | ¡ | ¡ |

17.	Has a doctor or other health care provider EVER told you that this child has…? *(continued)*

|  | Yes | No |
| --- | --- | --- |
| l. Other genetic or inherited condition | ¡ | ¡ |
| m. Heart failure | ¡ | ¡ |
| n. Other *(Please specify)* | ¡ | ¡ |

Does this child have any of the following?

|  | Yes | No |
| --- | --- | --- |
| a. Deafness or problems with hearing | ¡ | ¡ |
| b. Blindness or problems with seeing, even when wearing eyeglasses | ¡ | ¡ |
| *If your child is younger than 6 years old, go to question 19.* | | |
| c. Serious difficulty walking or climbing stairs | ¡ | ¡ |

**Your child’s healthcare needs**

Does this child CURRENTLY need or use medicine prescribed by a doctor, other than vitamins?

Yes

No

Does this child need or use more medical care, mental health, or educational services than is usual for most children of the same age?

Yes

No

Is this child limited or prevented in any way in their ability to do the things most children of the same age can do?

Yes

No

Go to question 23

To what extent do this child’s health conditions or problems affect their ability to do things?

Very little

Somewhat

A great deal

Does this child need or get special therapy, such as physical, occupational, or speech therapy?

Yes

No

Does this child have any kind of emotional, developmental, or behavioral problem for which they need treatment or counseling?

Yes

No

***If you answered “Yes” to any questions in this section (questions 19-24), continue to question 25. Otherwise, go to the next section.***

If **YES** to any of the questions in this section (questions 19-24), is it because of ANY medical, behavioral, or other health condition that is expected to last 12 months or longer?

Yes

No

**Your child’s school / learning history**

***If your child is younger than 3 years old, go to question 46.
If your child is between 3 and 5 years old, go to question 34.
Otherwise, continue to question 26.***

What grade is this child currently in? (If summer, what is the highest grade level this child has already completed?)

Kindergarten

1^st^ grade

2^nd^ grade

3^rd^ grade

4^th^ grade

5^th^ grade

6^th^ grade

7^th^ grade

8^th^ grade

9^th^ grade

10^th^ grade

11^th^ grade

12^th^ grade

Since starting kindergarten, has this child repeated any grades?

Yes

No

DURING THE PAST 12 MONTHS, about how many days did this child miss school because of their heart condition, illness, or injury?

No missed school days

1-3 days

4-6 days

7-10 days

11 or more days

This child was not enrolled in school

Has this child EVER had any of the following special education or early intervention plans? *(Select all that apply)*

Individualized Family Service Plan or IFSP (used for early intervention services in children younger than 3)

Individualized Education Program or IEP (used for special education services in children 3 or older)

504 Plan (sometimes used for special education services instead of or in addition to an IEP)

Other *(Please specify*)

No, this child has never had a plan for special education

***If your child is younger than 12 years old, go to question 31.
Otherwise, continue to question 30.***

How likely do you think it is that this child will…?

|  | Definitely Will | Probably Will | Probably Won’t | Definitely Won’t | Don’t Know | Already Has |
| --- | --- | --- | --- | --- | --- | --- |
| a. Get a regular high school diploma (A regular high school diploma includes a “GED” but does not include a certificate of completion or a special diploma for students in special education.) | ¡ | ¡ | ¡ | ¡ | ¡ | ¡ |
| b. Attend school after high school (including technical or trade school) | ¡ | ¡ | ¡ | ¡ | ¡ | ¡ |
| c. Attend a special training program after high school for persons with intellectual disabilities | ¡ | ¡ | ¡ | ¡ | ¡ | ¡ |
| d. Complete a technical or trade school program | ¡ | ¡ | ¡ | ¡ | ¡ | ¡ |
| e. Graduate from a 2-year or community college | ¡ | ¡ | ¡ | ¡ | ¡ | ¡ |

30.	How likely do you think it is that this child will…? *(continued)*

|  | Definitely Will | Probably Will | Probably Won’t | Definitely Won’t | Don’t Know | Already Has |
| --- | --- | --- | --- | --- | --- | --- |
| f. Graduate from a 4-year college | ¡ | ¡ | ¡ | ¡ | ¡ | ¡ |
| g. Get a driver’s license | ¡ | ¡ | ¡ | ¡ | ¡ | ¡ |
| h. Eventually live away from home on their own without supervision | ¡ | ¡ | ¡ | ¡ | ¡ | ¡ |
| i. Eventually live away on their own with supervision | ¡ | ¡ | ¡ | ¡ | ¡ | ¡ |
| j. Eventually get a paid job   (This includes any paid job -- child does not need to make enough to support self. This can include sheltered or supported employment.) | ¡ | ¡ | ¡ | ¡ | ¡ | ¡ |
| k. Earn enough to support themself without financial help from their family or government benefit programs | ¡ | ¡ | ¡ | ¡ | ¡ | ¡ |

**Your child’s activities and social environment**

DURING THE PAST WEEK, on how many days did this child exercise, play a sport, or participate in physical activity for at least 60 minutes?

0 days

1-3 days

4-6 days

Every day

DURING THE PAST 12 MONTHS, did this child participate in…?

|  | Yes | No |
| --- | --- | --- |
| a. A sports team or did they take sports lessons after school or on weekends | ¡ | ¡ |
| b. Any clubs or organizations after school or on weekends | ¡ | ¡ |
| c. Any other organized activities or lessons, such as music, dance, language, or other arts | ¡ | ¡ |
| d. Any type of community service or volunteer work at school, church, or in the community | ¡ | ¡ |
| e. Any work, including regular jobs as well as babysitting, cutting grass, or other occasional work | ¡ | ¡ |

DURING THE PAST 12 MONTHS, how often was this child bullied, picked on, or excluded by other children?

Never

1-2 times (in the past 12 months)

1-2 times per month

1-2 times per week

Almost every day

Compared to other children their age, how much difficulty does this child have making or keeping friends?

No difficulty

A little difficulty

A lot of difficulty

**Your child’s readiness to learn**

***If your child is between 3 and 5 years old, continue to question 35. Otherwise, go to question 46.***

How often does this child share toys or games with other children?

Always

Most of the time

About half of the time

Sometimes

Never

How often does this child show concern when they see others who are hurt or unhappy?

Always

Most of the time

About half of the time

Sometimes

Never

How often does this child play well with other children?

Always

Most of the time

About half of the time

Sometimes

Never

How often can this child recognize and name their own emotions?

Always

Most of the time

About half of the time

Sometimes

Never

How often does this child have difficulty when asked to end one activity and start a new activity?

Always

Most of the time

About half of the time

Sometimes

Never

How often does this child lose their temper?

Always

Most of the time

About half of the time

Sometimes

Never

How often does this child have trouble calming down?

Always

Most of the time

About half of the time

Sometimes

Never

How often does this child have difficulty waiting for their turn?

Always

Most of the time

About half of the time

Sometimes

Never

How often does this child get easily distracted?

Always

Most of the time

About half of the time

Sometimes

Never

How often can this child focus on a task you have given them for at least a few minutes? For example, simple chores?

Always

Most of the time

About half of the time

Sometimes

Never

How often does this child keep working at a task even when it is hard for them?

Always

Most of the time

About half of the time

Sometimes

Never

**Your child’s health care**

Where does this child usually go when they are sick or you need advice about their health?

This child does not have a usual place for health care or advice when sick

Doctor’s office

Hospital emergency room

Hospital outpatient department

Urgent care center

Clinic or health center

Retail store clinic or “Minute Clinic”

School (nurse’s office, athletic trainer’s office)

Some other place

DURING THE PAST 12 MONTHS, how many times did this child visit a doctor, nurse, or other health care professional to receive a PREVENTIVE check-up? A preventive check-up is when this child was not sick or injured, such as an annual or sports physical, or well-child visit.

0 visits

1 visit

2 or more visits

DURING THE PAST 12 MONTHS, how many times did this child visit a hospital emergency room?

None

1 time

2 or more times

DURING THE PAST 12 MONTHS, was this child admitted to the hospital to stay for at least one night?

Yes

No

**Your child’s unmet needs**

DURING THE PAST 12 MONTHS, did this child need any of the following health care but it was not received? *(Select all that apply)*

Heart care

Other medical care

Dental care

Vision care

Hearing care

Mental health services

Other *(Please specify)*

This child has received all the healthcare they needed
in the past 12 months

Go to question 52

Did any of the following reasons contribute to this child not receiving needed health services?

|  | Yes | No |
| --- | --- | --- |
| a. This child did not have health insurance that covered the services needed | ¡ | ¡ |
| b. This child was not eligible for the services | ¡ | ¡ |
| c. The services this child needed were not available in your area | ¡ | ¡ |
| d. There were problems getting an appointment when this child needed one | ¡ | ¡ |
| e. There were problems with getting transportation or child care | ¡ | ¡ |
| f. I had too many other things going on | ¡ | ¡ |
| g. The clinic or doctor’s office wasn’t open when this child needed care | ¡ | ¡ |
| h. There were issues related to cost | ¡ | ¡ |
| i. I chose to postpone or cancel appointments due to COVID-19 | ¡ | ¡ |
| j. The clinic or doctor’s office postponed or cancelled appointments due to COVID-19 | ¡ | ¡ |
| k. Other *(Please specify)* | ¡ | ¡ |

**Your child’s insurance**

Is this child CURRENTLY covered by ANY kind of health insurance or health coverage plan?

Yes

If your child is at least 12 years old, go to question 54.
Otherwise, go to question 60.

No

Is this child CURRENTLY covered by any of the following types of health insurance or health coverage plans?

|  | Yes | No |
| --- | --- | --- |
| a. Insurance through a current or former employer or union | ¡ | ¡ |
| b. Insurance purchased directly from an insurance company, including the Health Insurance Marketplace from the Affordable Care Act (ACA) | ¡ | ¡ |
| c. Medicaid (including MassHealth, MinnesotaCare, PeachCare, or Georgia Families), Medical Assistance, or any kind of government assistance plan for those with low incomes or a disability | ¡ | ¡ |
| d. TRICARE or other military health care | ¡ | ¡ |
| e. Indian Health Service | ¡ | ¡ |
| f. Other *(Please specify)* | ¡ | ¡ |

**Transition to adult healthcare**

***If your child is at least 12 years old, continue to question 54.
Otherwise, go to question 60.***

Eligibility for health insurance often changes in young adulthood. Do you know how this child will be insured as they become an adult?

Yes

Go to question 56

No

Has anyone discussed with you how to obtain or keep some type of health insurance coverage as this child becomes an adult?

Yes

No

Has a doctor or other health care provider ever discussed with you this child’s need to see a heart doctor throughout their life?

Yes

No

Has this child’s doctor or other health care provider actively worked with the child to:

|  | Yes | No | Don’t Know |
| --- | --- | --- | --- |
| a. Make positive choices about their health? For example, by eating healthy, getting regular exercise, not using tobacco, alcohol or other drugs, or delaying sexual activity. | ¡ | ¡ | ¡ |
| b. Gain skills to manage their health and health care? For example, by understanding current health needs, knowing what to do in a medical emergency, or taking medications they may need. | ¡ | ¡ | ¡ |
| c. Understand the changes in health care that happen at age 18? For example, by understanding changes in privacy, consent, access to information, or decision-making. | ¡ | ¡ | ¡ |

How prepared do you feel this child is to make positive choices about their health, manage their own health and health care, and handle changes in health care that happen at age 18?

Very prepared

Somewhat prepared

Not very prepared

Not at all prepared

Have this child’s doctors or other health care providers worked with you and this child to create a plan of care to meet their health goals and needs?

Yes

No

Please rate how concerned you are about this child’s future health.

Very concerned

Somewhat concerned

Not very concerned

Not at all concerned

**Immunizations**

DURING THE PAST 12 MONTHS, has this child had a flu vaccination? A flu vaccination is usually given in the fall and protects against influenza for the flu season.

Yes

No

**COVID-19**

Has this child ever had coronavirus or COVID-19 (based on a positive test for COVID-19 or a health professional telling you the child had COVID-19)?

Yes

No

Did not receive results

Please select the statement that best describes this child regarding the COVID-19 vaccine.

This child has received all recommended doses of
vaccine for COVID-19

Go to question 65

This child has received some but not all recommended
doses of vaccine, and I intend for them to receive all
recommended doses

This child has received some but not all recommended
doses of vaccine, and I do not intend for them to receive
all recommended doses

This child has not received any vaccine for COVID-19

Other

What are your reasons for choosing not to get this child fully vaccinated for COVID-19? *(Select all that apply)*

I’m concerned about the potential side effects of the vaccine

I feel the vaccines were created too quickly

I don’t believe the vaccines are effective at preventing the spread of COVID-19

I’m not concerned about this child contracting COVID-19

I’m generally opposed to vaccinations

A friend or family member had a bad reaction to the vaccine

I don’t think that a vaccine is necessary because COVID-19 is not a serious threat

This child’s doctor advised me not to get this child vaccinated

Other *(Please specify)*

I prefer not to say

**About you**

How are you related to this child?

Biological, adoptive, or stepparent

Foster parent

Other: Relative

Other: Non-relative

What is your age in years?

19 or younger

20-29

30-39

40-49

50-59

60-69

70 or older

What is the highest grade or level of school you have completed?

12th grade or less; no diploma

High school graduate or GED completed

College graduate or higher

Which of the following best describes your current employment status?

Employed full time

Employed part-time

Not employed or working WITHOUT pay

In general, how is your mental or emotional health?

Excellent

Very good

Good

Fair

Poor

How well do you feel that you are handling the day-to-day demands of raising a child with a heart condition?

Very well

Somewhat well

Not very well

Not well at all

DURING THE PAST 12 MONTHS, was there someone that you could turn to for day-to-day emotional support with parenting or raising a child with a heart condition?

Yes

No

Go to question 73

Did you receive support from…?

|  | Yes | No |
| --- | --- | --- |
| a. Spouse or domestic partner | ¡ | ¡ |
| b. Other family member or close friend | ¡ | ¡ |
| c. Health care provider | ¡ | ¡ |
| d. Place of worship or religious leader | ¡ | ¡ |
| e. Support or advocacy group related to specific health condition | ¡ | ¡ |
| f. Peer support group | ¡ | ¡ |
| g. Counselor or other mental health professional | ¡ | ¡ |
| h. Other person *(Please specify)* | ¡ | ¡ |

DURING THE PAST 12 MONTHS, did your family have problems paying for any of this child’s medical or health care bills?

Yes

No

DURING THE PAST 12 MONTHS, have you or other family members…?

|  | Yes | No |
| --- | --- | --- |
| a. Left a job or taken a leave of absence because of this child’s health or health conditions | ¡ | ¡ |
| b. Cut down on the hours you work because of this child’s health or health conditions | ¡ | ¡ |
| c. Avoided changing jobs because of concerns about maintaining health insurance for this child | ¡ | ¡ |

IN AN AVERAGE WEEK, how many hours do you or other family members spend providing health care at home for this child? *Care might include changing bandages or giving medication and therapies when needed.*

This child does not need health care provided at home on a weekly basis

Less than 1 hour per week

1-4 hours per week

5-10 hours per week

11 or more hours per week

At any time DURING THE PAST 12 MONTHS, even for one month, did anyone in your family receive…?

|  | Yes | No |
| --- | --- | --- |
| a. Cash assistance from a government welfare program | ¡ | ¡ |
| b. Food stamps or Supplemental Nutrition Assistance Program (SNAP) benefits | ¡ | ¡ |
| c. Free or reduced-cost breakfast or lunches at school | ¡ | ¡ |
| d. Benefits from the Women, Infants, and Children (WIC) program | ¡ | ¡ |

SINCE THIS CHILD WAS BORN, how often has it been very hard to cover the basics, like food or housing, on your family’s income?

Never

Rarely

Somewhat often

Very often

**Household information**

Does this child have another parent or adult caregiver who lives in this household?

Yes

No

Go to question 82

How is this other caregiver related to this child?

Biological adoptive, or stepparent

Foster parent

Other: Relative

Other: Non-relative

What is the highest grade or level of school this caregiver has completed?

12th grade or less; no diploma

High school graduate or GED completed

College graduate or higher

Which of the following best describes this caregiver’s current employment status?

Employed full time

Employed part-time

Not employed or working WITHOUT pay

How many children under the age of 18 are now living in the household, **not including this child**?

Numeric response:

What is the primary language spoken in the household?

English

Spanish

Somali

Other *(Please specify)*

**Childhood experiences**

The next questions are about events that may have happened during this child’s life. These things can happen in any family, but some people may feel uncomfortable with these questions. As a reminder, you may skip any questions you do not want to answer.

To the best of your knowledge, has this child EVER experienced any of the following?

|  | Yes | No |
| --- | --- | --- |
| a. Parent or guardian divorced or separated | ¡ | ¡ |
| b. Parent or guardian died | ¡ | ¡ |
| c. Parent or guardian served time in jail or prison | ¡ | ¡ |
| d. Saw or heard parents or adults slap, hit, kick, or punch one another in the home | ¡ | ¡ |
| e. Was a victim of violence or witnessed violence in their neighborhood | ¡ | ¡ |
| f. Lived with anyone who was mentally ill, suicidal, or severely depressed | ¡ | ¡ |
| g. Lived with anyone who had a problem with alcohol or drugs | ¡ | ¡ |
| h. Treated or judged unfairly because of their race or ethnic group | ¡ | ¡ |
| i. Treated or judged unfairly because of a health condition or disability | ¡ | ¡ |
| *If your child is younger than 6 years old, go to question 85.* | | |
| j. Treated or judged unfairly because of their sexual orientation or gender identity | ¡ | ¡ |

**Future needs**

What expectations do you have for this child in the future?

What concerns do you have for this child in the future?

**Contact information**

If you would like to receive periodic updates on the progress and results of this survey, please provide your email address.

***Please mail the survey back to us in the enclosed postage paid envelope.***

*Public reporting burden for this collection of information is estimated to average 20 minutes, including completing and reviewing the collection of information. An agency may not conduct or sponsor and a person is not required to respond to a collection of information unless it displays a currently valid OMB control number. Send comments regarding this burden estimate or any other aspect of this collection of information, including suggestions for reducing this burden to CDC/ATSDR Reports Clearance Officer, 1600 Clifton Road NE, MS D-74, Atlanta, GA 30333: ATTN: PRA (0920-22CL).*

##### **Appendix D. Descriptions of data sources used to incorporate geography-level socioeconomic and rurality measures for CHSTRONG KIDS cases**

| **RELEVANT MEASURE** | **GEOGRAPHY** | **DATA SOURCE** | **DATA YEAR(S) FOR LINKAGE TO ADDRESS AT BIRTH^1^** | **DATA YEAR(S) FOR LINKAGE TO ADDRESS AT SURVEY^2^** | **DATA COLLECTED** |
| --- | --- | --- | --- | --- | --- |
| Neighborhood Deprivation Index | Census Tract | United States Census Bureau - American Community Survey 5-year estimates    Website: https://www.census.gov/data/developers/data-sets/acs-5year.html | Cases born during or before 2010 are assigned values based on 2007-2011 ACS data, due to substantial missing data in 2006-2010 ACS.  For all other cases, assigned values are based on ACS 5-year estimates ending in year of birth (e.g. 2011 yob = 2007-2011 ACS; 2012 yob = 2008-2012 ACS; etc.). | 2023 | The Neighborhood Deprivation Index computed using data from all U.S. census tracts. Values range from ‐3.6 to +2.8. Higher values indicate more neighborhood deprivation (lower socioeconomic status) |
|  |  |  |  |  | Quintiles for the Neighborhood Deprivation Index |
|  |  |  |  |  | Median household income (dollars) SES dimension: wealth and income ACS table source: B19013 |
|  |  |  |  |  | Percent of households receiving dividends, interest, or rental income SES dimension: wealth and income ACS table source: B19054 |
|  |  |  |  |  | Percent of households receiving public assistance SES dimension: wealth and income ACS table source: B19058 |
|  |  |  |  |  | Median home value (dollars) SES dimension: wealth and income ACS table source: B25077 |
|  |  |  |  |  | Percent in a management, business, science, or arts occupation SES dimension: occupation ACS table source: C24060 |
|  |  |  |  |  | Percent of households that are female headed with any children under 18 SES dimension: housing conditions ACS table source: B11005 |
|  |  |  |  |  | Percent of housing units that are owner occupied SES dimension: housing conditions ACS table source: DP04 |
|  |  |  |  |  | Percent of households without a telephone SES dimension: housing conditions ACS table source: DP04 |
|  |  |  |  |  | Percent of households without complete plumbing facilities SES dimension: housing conditions ACS table source: DP04 |
|  |  |  |  |  | Percent with a high school degree or higher SES dimension: education ACS table source: S1501 |
|  |  |  |  |  | Percent with a college degree or higher SES dimension: education ACS table source: S1501 |
|  |  |  |  |  | Percent of families with incomes below the poverty level SES dimension: wealth and income ACS table source: S1702 |
|  |  |  |  |  | Percent unemployed SES dimension: occupation ACS table source: S2301 |
| Social Vulnerability Index | Census Tract | Centers for Disease Control and Prevention/ Agency for Toxic Substances and Disease Registry/ Geospatial Research, Analysis, and Services Program.   Website: https://www.atsdr.cdc.gov/place-health/php/svi/svi-data-documentation-download.html | 2010, 2014, 2016, 2018, and 2020 estimates collected for all cases | 2020 and 2022 collected for all survey respondents | Percentile ranking for Socioeconomic theme summary (State) |
|  |  |  |  |  | Percentile ranking for Household Composition theme summary (State) |
|  |  |  |  |  | Percentile ranking for Minority Status/Language theme (State) |
|  |  |  |  |  | Percentile ranking for Housing Type/Transportation theme (State) |
|  |  |  |  |  | Overall SVI percentile ranking (State) |
|  |  |  |  |  | Percentile ranking for Socioeconomic theme summary (National) |
|  |  |  |  |  | Percentile ranking for Household Composition theme summary (National) |
|  |  |  |  |  | Percentile ranking for Minority Status/Language theme (National) |
|  |  |  |  |  | Percentile ranking for Housing Type/Transportation theme (National) |
|  |  |  |  |  | Overall SVI percentile ranking (National) |
| Census Race | Census Tract | United States Census Bureau - Decennial Census (2010)  Website: https://www.census.gov/programs-surveys/decennial-census/decade.html | 2010 | N/A | Total |
|  |  |  |  |  | Errata of Total |
|  |  |  |  |  | Population of one race |
|  |  |  |  |  | Population of one race: White alone |
|  |  |  |  |  | Population of one race: Black or African American alone |
|  |  |  |  |  | Population of one race: American Indian and Alaska Native alone |
|  |  |  |  |  | Population of one race: Asian alone |
|  |  |  |  |  | Population of one race: Native Hawaiian and Other Pacific Islander alone |
|  |  |  |  |  | Population of one race: Some Other Race alone |
|  |  |  |  |  | Two or More Races |
|  |  |  |  |  | Population of two races |
|  |  |  |  |  | Population of two races: White; Black or African American |
|  |  |  |  |  | Population of two races: White; American Indian and Alaska Native |
|  |  |  |  |  | Population of two races: White; Asian |
|  |  |  |  |  | Population of two races: White; Native Hawaiian and Other Pacific Islander |
|  |  |  |  |  | Population of two races: White; Some Other Race |
|  |  |  |  |  | Population of two races: Black or African American; American Indian and Alaska Native |
|  |  |  |  |  | Population of two races: Black or African American; Asian |
|  |  |  |  |  | Population of two races: Black or African American; Native Hawaiian and Other Pacific Islander |
|  |  |  |  |  | Population of two races: Black or African American; Some Other Race |
|  |  |  |  |  | Population of two races: American Indian and Alaska Native; Asian |
|  |  |  |  |  | Population of two races: American Indian and Alaska Native; Native Hawaiian and Other Pacific Islander |
|  |  |  |  |  | Population of two races: American Indian and Alaska Native; Some Other Race |
|  |  |  |  |  | Population of two races: Asian; Native Hawaiian and Other Pacific Islander |
|  |  |  |  |  | Population of two races: Asian; Some Other Race |
|  |  |  |  |  | Population of two races: Native Hawaiian and Other Pacific Islander; Some Other Race |
|  |  |  |  |  | Population of three races |
|  |  |  |  |  | Population of three races: White; Black or African American; American Indian and Alaska Native |
|  |  |  |  |  | Population of three races: White; Black or African American; Asian |
|  |  |  |  |  | Population of three races: White; Black or African American; Native Hawaiian and Other Pacific Islander |
|  |  |  |  |  | Population of three races: White; Black or African American; Some Other Race |
|  |  |  |  |  | Population of three races: White; American Indian and Alaska Native; Asian |
|  |  |  |  |  | Population of three races: White; American Indian and Alaska Native; Native Hawaiian and Other Pacific Islander |
|  |  |  |  |  | Population of three races: White; American Indian and Alaska Native; Some Other Race |
|  |  |  |  |  | Population of three races: White; Asian; Native Hawaiian and Other Pacific Islander |
|  |  |  |  |  | Population of three races: White; Asian; Some Other Race |
|  |  |  |  |  | Population of three races: White; Native Hawaiian and Other Pacific Islander; Some Other Race |
|  |  |  |  |  | Population of three races: Black or African American; American Indian and Alaska Native; Asian |
|  |  |  |  |  | Population of three races: Black or African American; American Indian and Alaska Native; Native Hawaiian and Other Pacific Islander |
|  |  |  |  |  | Population of three races: Black or African American; American Indian and Alaska Native; Some Other Race |
|  |  |  |  |  | Population of three races: Black or African American; Asian; Native Hawaiian and Other Pacific Islander |
|  |  |  |  |  | Population of three races: Black or African American; Asian; Some Other Race |
|  |  |  |  |  | Population of three races: Black or African American; Native Hawaiian and Other Pacific Islander; Some Other Race |
|  |  |  |  |  | Population of three races: American Indian and Alaska Native; Asian; Native Hawaiian and Other Pacific Islander |
|  |  |  |  |  | Population of three races: American Indian and Alaska Native; Asian; Some Other Race |
|  |  |  |  |  | Population of three races: American Indian and Alaska Native; Native Hawaiian and Other Pacific Islander; Some Other Race |
|  |  |  |  |  | Population of three races: Asian; Native Hawaiian and Other Pacific Islander; Some Other Race |
|  |  |  |  |  | Population of four races |
|  |  |  |  |  | Population of four races: White; Black or African American; American Indian and Alaska Native; Asian |
|  |  |  |  |  | Population of four races: White; Black or African American; American Indian and Alaska Native; Native Hawaiian and Other Pacific Islander |
|  |  |  |  |  | Population of four races: White; Black or African American; American Indian and Alaska Native; Some Other Race |
|  |  |  |  |  | Population of four races: White; Black or African American; Asian; Native Hawaiian and Other Pacific Islander |
|  |  |  |  |  | Population of four races: White; Black or African American; Asian; Some Other Race |
|  |  |  |  |  | Population of four races: White; Black or African American; Native Hawaiian and Other Pacific Islander; Some Other Race |
|  |  |  |  |  | Population of four races: White; American Indian and Alaska Native; Asian; Native Hawaiian and Other Pacific Islander |
|  |  |  |  |  | Population of four races: White; American Indian and Alaska Native; Asian; Some Other Race |
|  |  |  |  |  | Population of four races: White; American Indian and Alaska Native; Native Hawaiian and Other Pacific Islander; Some Other Race |
|  |  |  |  |  | Population of four races: White; Asian; Native Hawaiian and Other Pacific Islander; Some Other Race |
|  |  |  |  |  | Population of four races: Black or African American; American Indian and Alaska Native; Asian; Native Hawaiian and Other Pacific Islander |
|  |  |  |  |  | Population of four races: Black or African American; American Indian and Alaska Native; Asian; Some Other Race |
|  |  |  |  |  | Population of four races: Black or African American; American Indian and Alaska Native; Native Hawaiian and Other Pacific Islander; Some Other Race |
|  |  |  |  |  | Population of four races: Black or African American; Asian; Native Hawaiian and Other Pacific Islander; Some Other Race |
|  |  |  |  |  | Population of four races: American Indian and Alaska Native; Asian; Native Hawaiian and Other Pacific Islander; Some Other Race |
|  |  |  |  |  | Population of five races |
|  |  |  |  |  | Population of five races: White; Black or African American; American Indian and Alaska Native; Asian; Native Hawaiian and Other Pacific Islander |
|  |  |  |  |  | Population of five races: White; Black or African American; American Indian and Alaska Native; Asian; Some Other Race |
|  |  |  |  |  | Population of five races: White; Black or African American; American Indian and Alaska Native; Native Hawaiian and Other Pacific Islander; Some Other Race |
|  |  |  |  |  | Population of five races: White; Black or African American; Asian; Native Hawaiian and Other Pacific Islander; Some Other Race |
|  |  |  |  |  | Population of five races: White; American Indian and Alaska Native; Asian; Native Hawaiian and Other Pacific Islander; Some Other Race |
|  |  |  |  |  | Population of five races: Black or African American; American Indian and Alaska Native; Asian; Native Hawaiian and Other Pacific Islander; Some Other Race |
|  |  |  |  |  | Population of six races |
|  |  |  |  |  | Population of six races: White; Black or African American; American Indian and Alaska Native; Asian; Native Hawaiian and Other Pacific Islander; Some Other Race |
| Census Race | Census Tract | United States Census Bureau - Decennial Census (2020)  Website: https://www.census.gov/programs-surveys/decennial-census/decade.html | 2020 | 2020 | Total |
|  |  |  |  |  | Annotation of Total |
|  |  |  |  |  | Total:: Population of one race |
|  |  |  |  |  | Annotation of Total:: Population of one race |
|  |  |  |  |  | Population of one race:White alone |
|  |  |  |  |  | Annotation of Population of one race:White alone |
|  |  |  |  |  | Population of one race:Black or African American alone |
|  |  |  |  |  | Annotation of Population of one race:Black or African American alone |
|  |  |  |  |  | Population of one race:American Indian and Alaska Native alone |
|  |  |  |  |  | Annotation of Population of one race:American Indian and Alaska Native alone |
|  |  |  |  |  | Population of one race:Asian alone |
|  |  |  |  |  | Annotation of Population of one race:Asian alone |
|  |  |  |  |  | Population of one race:Native Hawaiian and Other Pacific Islander alone |
|  |  |  |  |  | Annotation of Population of one race:Native Hawaiian and Other Pacific Islander alone |
|  |  |  |  |  | Population of one race:Some Other Race alone |
|  |  |  |  |  | Annotation of Population of one race:Some Other Race alone |
|  |  |  |  |  | Total:Population of two or more races |
|  |  |  |  |  | Annotation of Total:Population of two or more races |
|  |  |  |  |  | Population of two races |
|  |  |  |  |  | Annotation of Population of two races |
|  |  |  |  |  | Population of two races:White; Black or African American |
|  |  |  |  |  | Annotation of Population of two races:White; Black or African American |
|  |  |  |  |  | Population of two races:White; American Indian and Alaska Native |
|  |  |  |  |  | Annotation of Population of two races:White; American Indian and Alaska Native |
|  |  |  |  |  | Population of two races:White; Asian |
|  |  |  |  |  | Annotation of Population of two races:White; Asian |
|  |  |  |  |  | Population of two races:White; Native Hawaiian and Other Pacific Islander |
|  |  |  |  |  | Annotation of Population of two races:White; Native Hawaiian and Other Pacific Islander |
|  |  |  |  |  | Population of two races:White; Some Other Race |
|  |  |  |  |  | Annotation of Population of two races:White; Some Other Race |
|  |  |  |  |  | Population of two races:Black or African American; American Indian and Alaska Native |
|  |  |  |  |  | Annotation of Population of two races:Black or African American; American Indian and Alaska Native |
|  |  |  |  |  | Population of two races:Black or African American; Asian |
|  |  |  |  |  | Annotation of Population of two races:Black or African American; Asian |
|  |  |  |  |  | Population of two races:Black or African American; Native Hawaiian and Other Pacific Islander |
|  |  |  |  |  | Annotation of Population of two races:Black or African American; Native Hawaiian and Other Pacific Islander |
|  |  |  |  |  | Population of two races:Black or African American; Some Other Race |
|  |  |  |  |  | Annotation of Population of two races:Black or African American; Some Other Race |
|  |  |  |  |  | Population of two races:American Indian and Alaska Native; Asian |
|  |  |  |  |  | Annotation of Population of two races:American Indian and Alaska Native; Asian |
|  |  |  |  |  | Population of two races:American Indian and Alaska Native; Native Hawaiian and Other Pacific Islander |
|  |  |  |  |  | Annotation of Population of two races:American Indian and Alaska Native; Native Hawaiian and Other Pacific Islander |
|  |  |  |  |  | Population of two races:American Indian and Alaska Native; Some Other Race |
|  |  |  |  |  | Annotation of Population of two races:American Indian and Alaska Native; Some Other Race |
|  |  |  |  |  | Population of two races:Asian; Native Hawaiian and Other Pacific Islander |
|  |  |  |  |  | Annotation of Population of two races:Asian; Native Hawaiian and Other Pacific Islander |
|  |  |  |  |  | Population of two races:Asian; Some Other Race |
|  |  |  |  |  | Annotation of Population of two races:Asian; Some Other Race |
|  |  |  |  |  | Population of two races:Native Hawaiian and Other Pacific Islander; Some Other Race |
|  |  |  |  |  | Annotation of Population of two races:Native Hawaiian and Other Pacific Islander; Some Other Race |
|  |  |  |  |  | Population of three races |
|  |  |  |  |  | Annotation of Population of three races |
|  |  |  |  |  | Population of three races:White; Black or African American; American Indian and Alaska Native |
|  |  |  |  |  | Annotation of Population of three races:White; Black or African American; American Indian and Alaska Native |
|  |  |  |  |  | Population of three races:White; Black or African American; Asian |
|  |  |  |  |  | Annotation of Population of three races:White; Black or African American; Asian |
|  |  |  |  |  | Population of three races:White; Black or African American; Native Hawaiian and Other Pacific Islander |
|  |  |  |  |  | Annotation of Population of three races:White; Black or African American; Native Hawaiian and Other Pacific Islander |
|  |  |  |  |  | Population of three races:White; Black or African American; Some Other Race |
|  |  |  |  |  | Annotation of Population of three races:White; Black or African American; Some Other Race |
|  |  |  |  |  | Population of three races:White; American Indian and Alaska Native; Asian |
|  |  |  |  |  | Annotation of Population of three races:White; American Indian and Alaska Native; Asian |
|  |  |  |  |  | Population of three races:White; American Indian and Alaska Native; Native Hawaiian and Other Pacific Islander |
|  |  |  |  |  | Annotation of Population of three races:White; American Indian and Alaska Native; Native Hawaiian and Other Pacific Islander |
|  |  |  |  |  | Population of three races:White; American Indian and Alaska Native; Some Other Race |
|  |  |  |  |  | Annotation of Population of three races:White; American Indian and Alaska Native; Some Other Race |
|  |  |  |  |  | Population of three races:White; Asian; Native Hawaiian and Other Pacific Islander |
|  |  |  |  |  | Annotation of Population of three races:White; Asian; Native Hawaiian and Other Pacific Islander |
|  |  |  |  |  | Population of three races:White; Asian; Some Other Race |
|  |  |  |  |  | Annotation of Population of three races:White; Asian; Some Other Race |
|  |  |  |  |  | Population of three races:White; Native Hawaiian and Other Pacific Islander; Some Other Race |
|  |  |  |  |  | Annotation of Population of three races:White; Native Hawaiian and Other Pacific Islander; Some Other Race |
|  |  |  |  |  | Population of three races:Black or African American; American Indian and Alaska Native; Asian |
|  |  |  |  |  | Annotation of Population of three races:Black or African American; American Indian and Alaska Native; Asian |
|  |  |  |  |  | Population of three races:Black or African American; American Indian and Alaska Native; Native Hawaiian and Other Pacific Islander |
|  |  |  |  |  | Annotation of Population of three races:Black or African American; American Indian and Alaska Native; Native Hawaiian and Other Pacific Islander |
|  |  |  |  |  | Population of three races:Black or African American; American Indian and Alaska Native; Some Other Race |
|  |  |  |  |  | Annotation of Population of three races:Black or African American; American Indian and Alaska Native; Some Other Race |
|  |  |  |  |  | Population of three races:Black or African American; Asian; Native Hawaiian and Other Pacific Islander |
|  |  |  |  |  | Annotation of Population of three races:Black or African American; Asian; Native Hawaiian and Other Pacific Islander |
|  |  |  |  |  | Population of three races:Black or African American; Asian; Some Other Race |
|  |  |  |  |  | Annotation of Population of three races:Black or African American; Asian; Some Other Race |
|  |  |  |  |  | Population of three races:Black or African American; Native Hawaiian and Other Pacific Islander; Some Other Race |
|  |  |  |  |  | Annotation of Population of three races:Black or African American; Native Hawaiian and Other Pacific Islander; Some Other Race |
|  |  |  |  |  | Population of three races:American Indian and Alaska Native; Asian; Native Hawaiian and Other Pacific Islander |
|  |  |  |  |  | Annotation of Population of three races:American Indian and Alaska Native; Asian; Native Hawaiian and Other Pacific Islander |
|  |  |  |  |  | Population of three races:American Indian and Alaska Native; Asian; Some Other Race |
|  |  |  |  |  | Annotation of Population of three races:American Indian and Alaska Native; Asian; Some Other Race |
|  |  |  |  |  | Population of three races:American Indian and Alaska Native; Native Hawaiian and Other Pacific Islander; Some Other Race |
|  |  |  |  |  | Annotation of Population of three races:American Indian and Alaska Native; Native Hawaiian and Other Pacific Islander; Some Other Race |
|  |  |  |  |  | Population of three races:Asian; Native Hawaiian and Other Pacific Islander; Some Other Race |
|  |  |  |  |  | Annotation of Population of three races:Asian; Native Hawaiian and Other Pacific Islander; Some Other Race |
|  |  |  |  |  | Population of four races |
|  |  |  |  |  | Annotation of Population of four races |
|  |  |  |  |  | Population of four races:White; Black or African American; American Indian and Alaska Native; Asian |
|  |  |  |  |  | Annotation of Population of four races:White; Black or African American; American Indian and Alaska Native; Asian |
|  |  |  |  |  | Population of four races:White; Black or African American; American Indian and Alaska Native; Native Hawaiian and Other Pacific Islander |
|  |  |  |  |  | Annotation of Population of four races:White; Black or African American; American Indian and Alaska Native; Native Hawaiian and Other Pacific Islander |
|  |  |  |  |  | Population of four races:White; Black or African American; American Indian and Alaska Native; Some Other Race |
|  |  |  |  |  | Annotation of Population of four races:White; Black or African American; American Indian and Alaska Native; Some Other Race |
|  |  |  |  |  | Population of four races:White; Black or African American; Asian; Native Hawaiian and Other Pacific Islander |
|  |  |  |  |  | Annotation of Population of four races:White; Black or African American; Asian; Native Hawaiian and Other Pacific Islander |
|  |  |  |  |  | Population of four races:White; Black or African American; Asian; Some Other Race |
|  |  |  |  |  | Annotation of Population of four races:White; Black or African American; Asian; Some Other Race |
|  |  |  |  |  | Population of four races:White; Black or African American; Native Hawaiian and Other Pacific Islander; Some Other Race |
|  |  |  |  |  | Annotation of Population of four races:White; Black or African American; Native Hawaiian and Other Pacific Islander; Some Other Race |
|  |  |  |  |  | Population of four races:White; American Indian and Alaska Native; Asian; Native Hawaiian and Other Pacific Islander |
|  |  |  |  |  | Annotation of Population of four races:White; American Indian and Alaska Native; Asian; Native Hawaiian and Other Pacific Islander |
|  |  |  |  |  | Population of four races:White; American Indian and Alaska Native; Asian; Some Other Race |
|  |  |  |  |  | Annotation of Population of four races:White; American Indian and Alaska Native; Asian; Some Other Race |
|  |  |  |  |  | Population of four races:White; American Indian and Alaska Native; Native Hawaiian and Other Pacific Islander; Some Other Race |
|  |  |  |  |  | Annotation of Population of four races:White; American Indian and Alaska Native; Native Hawaiian and Other Pacific Islander; Some Other Race |
|  |  |  |  |  | Population of four races:White; Asian; Native Hawaiian and Other Pacific Islander; Some Other Race |
|  |  |  |  |  | Annotation of Population of four races:White; Asian; Native Hawaiian and Other Pacific Islander; Some Other Race |
|  |  |  |  |  | Population of four races:Black or African American; American Indian and Alaska Native; Asian; Native Hawaiian and Other Pacific Islander |
|  |  |  |  |  | Annotation of Population of four races:Black or African American; American Indian and Alaska Native; Asian; Native Hawaiian and Other Pacific Islander |
|  |  |  |  |  | Population of four races:Black or African American; American Indian and Alaska Native; Asian; Some Other Race |
|  |  |  |  |  | Annotation of Population of four races:Black or African American; American Indian and Alaska Native; Asian; Some Other Race |
|  |  |  |  |  | Population of four races:Black or African American; American Indian and Alaska Native; Native Hawaiian and Other Pacific Islander; Some Other Race |
|  |  |  |  |  | Annotation of Population of four races:Black or African American; American Indian and Alaska Native; Native Hawaiian and Other Pacific Islander; Some Other Race |
|  |  |  |  |  | Population of four races:Black or African American; Asian; Native Hawaiian and Other Pacific Islander; Some Other Race |
|  |  |  |  |  | Annotation of Population of four races:Black or African American; Asian; Native Hawaiian and Other Pacific Islander; Some Other Race |
|  |  |  |  |  | Population of four races:American Indian and Alaska Native; Asian; Native Hawaiian and Other Pacific Islander; Some Other Race |
|  |  |  |  |  | Annotation of Population of four races:American Indian and Alaska Native; Asian; Native Hawaiian and Other Pacific Islander; Some Other Race |
|  |  |  |  |  | Population of five races |
|  |  |  |  |  | Annotation of Population of five races |
|  |  |  |  |  | Population of five races:White; Black or African American; American Indian and Alaska Native; Asian; Native Hawaiian and Other Pacific Islander |
|  |  |  |  |  | Annotation of Population of five races:White; Black or African American; American Indian and Alaska Native; Asian; Native Hawaiian and Other Pacific Islander |
|  |  |  |  |  | Population of five races:White; Black or African American; American Indian and Alaska Native; Asian; Some Other Race |
|  |  |  |  |  | Annotation of Population of five races:White; Black or African American; American Indian and Alaska Native; Asian; Some Other Race |
|  |  |  |  |  | Population of five races:White; Black or African American; American Indian and Alaska Native; Native Hawaiian and Other Pacific Islander; Some Other Race |
|  |  |  |  |  | Annotation of Population of five races:White; Black or African American; American Indian and Alaska Native; Native Hawaiian and Other Pacific Islander; Some Other Race |
|  |  |  |  |  | Population of five races:White; Black or African American; Asian; Native Hawaiian and Other Pacific Islander; Some Other Race |
|  |  |  |  |  | Annotation of Population of five races:White; Black or African American; Asian; Native Hawaiian and Other Pacific Islander; Some Other Race |
|  |  |  |  |  | Population of five races:White; American Indian and Alaska Native; Asian; Native Hawaiian and Other Pacific Islander; Some Other Race |
|  |  |  |  |  | Annotation of Population of five races:White; American Indian and Alaska Native; Asian; Native Hawaiian and Other Pacific Islander; Some Other Race |
|  |  |  |  |  | Population of five races:Black or African American; American Indian and Alaska Native; Asian; Native Hawaiian and Other Pacific Islander; Some Other Race |
|  |  |  |  |  | Annotation of Population of five races:Black or African American; American Indian and Alaska Native; Asian; Native Hawaiian and Other Pacific Islander; Some Other Race |
|  |  |  |  |  | Population of six races |
|  |  |  |  |  | Annotation of Population of six races |
|  |  |  |  |  | Population of six races:White; Black or African American; American Indian and Alaska Native; Asian; Native Hawaiian and Other Pacific Islander; Some Other Race |
|  |  |  |  |  | Annotation of Population of six races:White; Black or African American; American Indian and Alaska Native; Asian; Native Hawaiian and Other Pacific Islander; Some Other Race |
| Rural-Urban Continuum Codes (RUCC) | County | United States Department of Agriculture - Rural-Urban Continuum Codes  Website: https://www.ers.usda.gov/data-products/rural-urban-continuum-codes | 2013 and 2023 estimates collected for all cases | 2023 | RUCC Code |
| Rural-Urban Commuting Area (RUCA) | Census Tract | United States Department of Agriculture - Rural-Urban Commuting Area Codes  Website: https://www.ers.usda.gov/data-products/rural-urban-commuting-area-codes | 2010 collected for all cases | 2010 | Primary RUCA Code |
|  |  |  |  |  | Secondary RUCA Code |

CHD: congenital heart defects; CHSTRONG KIDS: Congenital Heart Survey To Recognize Outcomes, Needs, and well-beinG of KIDS

^1^Data were linked to address at birth for all CHD cases ascertained from birth defect surveillance systems for CHSTRONG KIDS

^2^Data were linked to address at survey for children whose parents or caregivers completed a CHSTRONG KIDS survey

**Appendix E. Methods for classifying cardiology follow-up (LTCF) timeframes**

The following table describes the methods for classifying cardiology follow-up timeframes in CHSTRONG KIDS, originally designed and implemented by Andrews et al.:

Andrews JG, Strah D, Downing KF, Kern MC, Oster ME, Seckeler MD, Goudie A, Nembhard WN, Farr SL, Klewer SE. Cardiology Care and Loss to Follow-Up Among Adults With Congenital Heart Defects in CH STRONG. Am J Cardiol. 2023 Jun 15;197:42-45. doi: 10.1016/j.amjcard.2023.04.004.

| BPA CODE | Condition | Chapter | NYHA Class A Follow-Up | Corresponding CHSTRONG KIDS response:  Time since last cardiology visit |
| --- | --- | --- | --- | --- |
| 745000 | Persistent truncus arteriosus | 4.4.4, 4.3.6 | 1y-2y | 1-2 years or sooner |
| 745010 | Aortic septal defect |  | None | Cannot classify |
| 745100 | Transposition of great vessels, complete (no VSD) | 4.4.1.1 | 1y | Less than 1 year |
| 745110 | Transposition of great vessels, incomplete (w/ VSD), Taussig Bing | 4.4.1.1 | 1y | Less than 1 year |
| 745120 | Corrected transposition of great vessels, L transposition, vent inversion | 4.4.1.4 | 1y-2y | 1-2 years or sooner |
| 745130 | Double outlet right ventricle - normally related great vessels | 4.4.1.1 | 1y | Less than 1 year |
| 745140 | Double outlet right ventricle - transposed great vessels | 4.4.1.1 | 1y | Less than 1 year |
| 745150 | Double outlet right ventricle - relationship of great vessels NOS | 4.4.1.1 | 1y | Less than 1 year |
| 745180 | Transposition of great vessels (TGA, TGV) - Other specified | 4.4.1.1 | 1y | Less than 1 year |
| 745190 | Unspecified transposition of great vessels | 4.4.1.1 | 1y | Less than 1 year |
| 745200 | Tetralogy of Fallot | 4.3.5 | 1y-2y | 1-2 years or sooner |
| 745210 | Fallot’s pentalogy (tetralogy plus ASD) | 4.3.5 | 1y-2y | 1-2 years or sooner |
| 745300 | Single ventricle, not specified | 4.4.2 | 1y | Less than 1 year |
| 745310 | Single ventricle, Double Inlet Left Ventricle | 4.4.2 | 1y | Less than 1 year |
| 745320 | Single ventricle, Double Inlet Right Ventricle | 4.4.2 | 1y | Less than 1 year |
| 745330 | Single ventricle, Indeterminate Ventricular Morphology | 4.4.2 | 1y | Less than 1 year |
| 745380 | Single ventricle, Other Specified | 4.4.2 | 1y | Less than 1 year |
| 745400 | Roger's disease | 4.1.3 | 3y-5y | 3-5 years or sooner |
| 745410 | Eisenmenger syndrome | 4.4.6 | 6m-1y | Less than 1 year |
| 745420 | Gerbode defect | 4.1.3 | 3y-5y | 3-5 years or sooner |
| 745480 | Ventricular septal defect (VSD) - Other specified | 4.1.3 | 3y-5y | 3-5 years or sooner |
| 745485 | Other specified ventricular septal defect | 4.1.3 | 3y-5y | 3-5 years or sooner |
| 745486 | Ventricular septal defect - Perimembranous or membranous | 4.1.3 | 3y-5y | 3-5 years or sooner |
| 745487 | Ventricular septal defect - Muscular, mid-muscular, apical | 4.1.3 | 3y-5y | 3-5 years or sooner |
| 745490 | Ventricular septal defect - Inlet | 4.1.3 | 3y-5y | 3-5 years or sooner |
| 745490 | Ventricular septal defect, NOS | 4.1.3 | 3y-5y | 3-5 years or sooner |
| 745510 | Atrial septal defect (ASD) - secundum | 4.1.1 | 3y-5y | 3-5 years or sooner |
| 745580 | Other specified atrial septal defect | 4.1.1 | 3y-5y | 3-5 years or sooner |
| 745590 | Atrial septal defect, NOS | 4.1.1 | 3y-5y | 3-5 years or sooner |
| 745520 | Lutembacher's syndrome | 4.1.1 | 3y-5y | 3-5 years or sooner |
| 745580 | Atrial septal defect, Other specified | 4.1.1 | 3y-5y | 3-5 years or sooner |
| 745590 | Atrial septal defect, NOS | 4.1.1 | 3y-5y | 3-5 years or sooner |
| 745600 | Ostium primum defects | 4.1.1 | 3y-5y | 3-5 years or sooner |
| 745610 | Single common atrium | 4.1.1 | 3y-5y | 3-5 years or sooner |
| 745620 | Common atrioventricular canal with ventricular septal defect | 4.1.4 | 2y-3y | 3-5 years or sooner |
| 745630 | Common atrioventricular canal, no mention of ventricular septal defect | 4.1.4 | 2y-3y | 3-5 years or sooner |
| 745680 | Other specified cushion defect | 4.1.4 | 2y-3y | 3-5 years or sooner |
| 745690 | Endocardial cushion defect | 4.1.4 | 2y-3y | 3-5 years or sooner |
| 745700 | Cor biloculare |  |  | Cannot classify |
| 745800 | Other bulbus cordis anomalies and anomalies of cardiac septal closure | 4.1.1, 4.1.3, 4.1.4 | 2y-3y-5y | 3-5 years or sooner |
| 745900 | Unspecified defect of septal closure | 4.1.1, 4.1., 4.1.4 | 2y-3y-5y | 3-5 years or sooner |
| 746000 | Pulmonary valve atresia, hypoplasia | 4.4.2 | 1y | Less than 1 year |
| 746010 | Pulmonary valve - Stenosis, congenital | 4.3.1 | 3y-5y | 3-5 years or sooner |
| 746030 | Pulmonary valve atresia with VSD | 4.4.2 | 1y | Less than 1 year |
| 746080 | Other specified anomalies of the pulmonary valve |  | None | Cannot classify |
| 746090 | Unspecified anomaly of pulmonary valve |  | None | Cannot classify |
| 746100 | Tricuspid valve atresia and stenosis | 4.4.2 | 1y | Less than 1 year |
| 746106 | Tricuspid valve - Stenosis or hypoplasia, congenital | 4.4.2 | 1y | Less than 1 year |
| 746200 | Ebstein's anomaly | 4.3.4 | 1y-2y | 1-2 years or sooner |
| 746300 | Aortic valve stenosis (includes valvar and subvalvar) | 4.2.3 | 2y | 1-2 years or sooner |
| 746480 | Other specified anomalies of aortic valve (including aortic valve atresia) | 4.2.3 | 2y | 1-2 years or sooner |
| 746490 | Unspecified anomalies of aortic valve | 4.2.3 | 2y | 1-2 years or sooner |
| 746500 | Congenital mitral stenosis | 4.2.2 | 2y | 1-2 years or sooner |
| 746505 | Absence, atresia, or hypoplasia of mitral valve |  |  | Cannot classify |
| 746700 | Hypoplastic left heart syndrome (HLHS) | 4.4.3, 4.4.2 | 1y | Less than 1 year |
| 746820 | Cor triatriatum | 4.2.1 | None | Cannot classify |
| 746830 | Pulmonary infundibular (subvalvular) stenosis | 4.2.3 | 2y | 1-2 years or sooner |
| 746840 | Trilogy of Fallot |  |  | Cannot classify |
| 746881 | Hypoplastic left ventricle | 4.4.2 | 1y | Less than 1 year |
| 746882 | Hypoplastic right heart/ ventricle (Uhl's disease) | 4.4.2 | 1y | Less than 1 year |
| 746883 | Hypoplastic ventricle, NOS | 4.4.2 | 1y | Less than 1 year |
| 746885 | Coronary artery anomaly | 4.4.2 | 1y | Less than 1 year |
| 746887 | Other defects of the atria |  | None | Cannot classify |
| 746900 | Unspecified anomalies of heart valves |  | None | Cannot classify |
| 746910 | Anomalous bands of heart |  | None | Cannot classify |
| 746920 | Acyanotic congenital heart disease, NOS |  | None | Cannot classify |
| 746930 | Cyanotic congenital heart disease, NOS |  | None | Cannot classify |
| 746995 | Pulmonic or pulmonary atresia, stenosis, or hypoplasia, NOS | 4.4.2 | 1y | Less than 1 year |
| 747100 | Coarctation of aorta, pre-ductal | 4.2.6 | 2y | 1-2 years or sooner |
| 747110 | Coarctation of aorta, post-ductal | 4.2.6 | 2y | 1-2 years or sooner |
| 747120 | Coarctation of aorta, ductal | 4.2.6 | 2y | 1-2 years or sooner |
| 747190 | Coarctation of aorta, NOS | 4.2.6 | 2y | 1-2 years or sooner |
| 747200 | Aorta atresia |  | None | Cannot classify |
| 747210 | Aorta hypoplasia |  | None | Cannot classify |
| 747215 | Interrupted aortic arch - Type A |  | None | Cannot classify |
| 747216 | Interrupted aortic arch - Type B |  | None | Cannot classify |
| 747217 | Interrupted aortic arch - Type C |  | None | Cannot classify |
| 747220 | Supra-aortic stenosis | 4.2.5 | 2y | 1-2 years or sooner |
| 747230 | Persistent right aortic arch |  | None | Cannot classify |
| 747240 | Aneurysm of sinus of Valsalva | 4.2.4 | None | Cannot classify |
| 747250 | Vascular ring (aorta) |  | None | Cannot classify |
| 747260 | Overriding aorta |  | None | Cannot classify |
| 747270 | Aortic aneurysm | 4.2.4 | None | Cannot classify |
| 747280 | Other specified anomalies of the aorta |  | None | Cannot classify |
| 747285 | Interrupted aortic arch, NOS |  | None | Cannot classify |
| 747290 | Aorta - Unspecified anomalies |  | None | Cannot classify |
| 747300 | Pulmonary artery atresia, absence, or agenesis | 4.3.1 | 3y-5y | 3-5 years or sooner |
| 747310 | Pulmonary artery - Atresia with septal defect | 4.3.1 | 3y-5y | 3-5 years or sooner |
| 747400 | Stenosis of vena cava (inferior or superior) | 4.1.2 | None | Cannot classify |
| 747410 | Persistent left superior vena cava | 4.1.2 | None | Cannot classify |
| 747420 | Total anomalous pulmonary venous return (TAPVR) | 4.1.2 | None | Cannot classify |
| 747430 | Partial anomalous pulmonary venous return (PAPVR) | 4.1.2 | None | Cannot Classify |
