# Supplementary material for: Characteristics of children with congenital heart defects and their caregivers in three U.S. sites: the Congenital Heart Survey To Recognize Outcomes Needs and well-beinG of KIDS (CHSTRONG KIDS)
Source: Prog Pediatr Cardiol. Author manuscript; Available in PMC 2026 Sep 19. (PMC13588542; doi:10.1016/j.ppedcard.2026.101956)
Supplement: Supplementary file 2 [file NIHMS2198443-supplement-Supplemental_Tables_and_Figures.docx]

**Supplemental Material.**

*Contents*

Supplemental Figure 1. Identification and recruitment of participants for CHSTRONG KIDS by site.

Supplemental Table 1. Characteristics at birth of children born 2006-2021 with CHD in Atlanta, Georgia, Massachusetts, and Minnesota by site, CHSTRONG KIDS – limited to heart defect diagnoses collected by all 3 sites

Supplemental Table 2. Characteristics at birth of children born 2006-2021 with CHD in Atlanta, Georgia, Massachusetts, and Minnesota by site and survey response status, CHSTRONG KIDS

Supplemental Table 3. Characteristics at birth among children eligible for CHSTRONG KIDS by survey response status – limited to heart defect diagnoses collected by all 3 sites

Supplemental Figure 2. Percentage of children with select types of severe CHD who were not up-to-date on their cardiology care, CHSTRONG KIDS – limited to heart defect diagnoses collected by all 3 sites

**Supplemental Figure 1. Identification and recruitment of participants for CHSTRONG KIDS by site.**

CHD: Congenital heart defects, CHSTRONG KIDS: Congenital Heart Survey To Recognize Outcomes, Needs, and well-beinG of KIDS, ATL= Atlanta, Georgia, MAS = Massachusetts, MIN: Minnesota

**Supplemental Table 1. Characteristics at birth of children born 2006-2021 with CHD in Atlanta, Georgia, Massachusetts, and Minnesota by site, CHSTRONG KIDS – limited to heart defect diagnoses collected by all 3 sites**

| **Characteristic** | **Overall n(%)**  (N = 3103) | | **ATL n(%)** (N = 677) | | **MAS n(%)** (N = 1344) | | **MIN n(%)** (N = 1082) | **p-value***^a^* | |
| --- | --- | --- | --- | --- | --- | --- | --- | --- | --- |
| Sex |  | |  | |  | |  | 0.76 | |
| Male | 1,843 (59.4%) | | 410 (60.6%) | | 797 (59.3%) | | 636 (58.8%) |  | |
| Female | 1,260 (40.6%) | | 267 (39.4%) | | 547 (40.7%) | | 446 (41.2%) |  | |
| Unknown | 0 | | 0 | | 0 | | 0 |  | |
| Year of birth |  | |  | |  | |  | <0.001 | |
| 2006-2011 | 1,123 (36.2%) | | 345 (51.0%) | | 495 (36.8%) | | 283 (26.2%) |  | |
| 2012-2017 | 1,208 (38.9%) | | 224 (33.1%) | | 523 (38.9%) | | 461 (42.6%) |  | |
| 2018-2021 | 772 (24.9%) | | 108 (16.0%) | | 326 (24.3%) | | 338 (31.2%) |  | |
| Gestational age at birth | | | | |  | |  | 0.65 | |
| Preterm (<37 weeks) | 484  (15.7%) | | 107  (16.1%) | | 201  (15.0%) | | 176  (16.3%) |  | |
| Full-term (≥37 weeks) | 2599  (84.3%) | | 556  (83.9%) | | 1138  (85.0%) | | 905  (83.7%) |  | |
| Unknown | 20 | | 14 | | 5 | | 1 |  | |
| Birth weight (grams) |  | |  | |  | |  | 0.39 | |
| <2500 (low) | 528 (17.2%) | | 115 (17.3%) | | 214 (16.3%) | | 199 (18.4%) |  | |
| ≥2500 (normal) | 2,534 (82.8%) | | 550 (82.7%) | | 1,101 (83.7%) | | 883 (81.6%) |  | |
| Unknown | 41 | | 12 | | 29 | | 0 |  | |
| Plurality |  | |  | |  | |  | 0.49 | |
| Singleton | 2,914 (94.7%) | | 618 (94.8%) | | 1,265 (94.2%) | | 1,031 (95.3%) |  | |
| Multiple birth | 163 (5.3%) | | 34 (5.2%) | | 78 (5.8%) | | 51 (4.7%) |  | |
| Unknown | 26 | | 25 | | 1 | | 0 |  | |
| CHD Severity |  | |  | |  | |  |  | |
| Severe | 3,103 (100.0%) | | 677 (100.0%) | | 1,344 (100.0%) | | 1,082 (100.0%) |  | |
| Shunt+Valve | 0 | | 0 | | 0 | | 0 |  | |
| Shunt | 0 | | 0 | | 0 | | 0 |  | |
| Valve | 0 | | 0 | | 0 | | 0 |  | |
| Other | 0 | | 0 | | 0 | | 0 |  | |
| Had a cardiac intervention in the first year of life*^b^* | | | | | | |  | <0.001 | |
| Yes | 1,883 (82.1%) | | NA | | 940 (70.0%) | | 943 (99.2%) |  | |
| No | 411  (17.9%) | | NA | | 403  (30.0) | | <10*^c^* |  | |
| Unknown | 132 | | NA | | 1 | | 131 |  | |
| Diagnosed with Trisomy 21 | | | | |  | |  | 0.002 | |
| Yes | 107 (3.4%) | | 10 (1.5%) | | 60 (4.5%) | | 37 (3.4%) |  | |
| No | 2996  (96.6%) | | 667  (98.5%) | | 1,284  (95.5%) | | 1,045  (96.6%) |  | |
| Mother’s marital status at delivery | |  | |  | |  | | <0.001 |  |
| Married | 1,803 (66.8%) | | 212 (58.9%) | | 837 (66.6%) | | 754 (69.7%) |  | |
| Unmarried | 895 (33.2%) | | 148 (41.1%) | | 419 (33.4%) | | 328 (30.3%) |  | |
| Unknown | 405 | | 317 | | 88 | | 0 |  | |
| Mother’s age at delivery (years) | |  | |  | |  | | <0.001 |  |
| ≤18 | 65 (2.1%) | | 21 (3.1%) | | 29 (2.2%) | | 15 (1.4%) |  | |
| 19-24 | 480 (15.5%) | | 122 (18.2%) | | 194 (14.4%) | | 164 (15.2%) |  | |
| 25-34 | 1,827 (59.0%) | | 379 (56.6%) | | 756 (56.3%) | | 692 (64.0%) |  | |
| ≥35 | 724 (23.4%) | | 148 (22.1%) | | 365 (27.2%) | | 211 (19.5%) |  | |
| Unknown | 7 | | 7 | | 0 | | 0 |  | |
| Mother’s educational attainment at delivery | | | | |  | |  | <0.001 | |
| Less than high school | 305 (10.3%) | | 72 (12.4%) | | 131 (9.9%) | | 102 (9.6%) |  | |
| High school diploma or equivalent | 612 (20.6%) | | 151 (26.0%) | | 245 (18.5%) | | 216 (20.3%) |  | |
| Some college, no degree | 469 (15.8%) | | 80 (13.8%) | | 193 (14.6%) | | 196 (18.4%) |  | |
| Associate degree | 274 (9.2%) | | 39 (6.7%) | | 138 (10.4%) | | 97 (9.1%) |  | |
| Bachelor’s degree | 797 (26.8%) | | 167 (28.7%) | | 351 (26.6%) | | 279 (26.2%) |  | |
| Graduate degree | 512 (17.2%) | | 72 (12.4%) | | 264 (20.0%) | | 176 (16.5%) |  | |
| Unknown | 134 | | 96 | | 22 | | 16 |  | |
| Mother’s race and ethnicity | |  | |  | |  | | <0.001 |  |
| Non-Hispanic White | 1,889 (61.9%) | | 237 (35.9%) | | 853 (64.6%) | | 799 (74.7%) |  | |
| Non-Hispanic Black | 496 (16.3%) | | 264 (39.9%) | | 122 (9.2%) | | 110 (10.3%) |  | |
| Hispanic | 409 (13.4%) | | 107 (16.2%) | | 233 (17.7%) | | 69 (6.4%) |  | |
| Non-Hispanic Native American or Alaskan Native | 14 (0.5%) | | 0 (0.0%) | | 0 (0.0%) | | 14 (1.3%) |  | |
| Non-Hispanic Asian or Pacific Islander | 217 (7.1%) | | 47 (7.1%) | | 95 (7.2%) | | 75 (7.0%) |  | |
| Non-Hispanic Other | 26 (0.9%) | | <10*^c^* | | 17 (1.3%) | | <10*^c^* |  | |
| Unknown | 52 | | 16 | | 24 | | 12 |  | |
| Rurality of birth residence |  | |  | |  | |  | <0.001 | |
| Urban | 2,927 (94.4%) | | 676  (100.0%) | | 1,328 (98.9%) | | 923  (85.3%) |  | |
| Rural | 174  (5.6%) | | 0  (0.0%) | | 15  (1.1%) | | 159  (14.7%) |  | |
| Unknown | 2 | | 1 | | 1 | | 0 |  | |
| ATL: Atlanta site, CHSTRONG KIDS: Congenital Heart Survey To Recognize Outcomes, Needs, and well-beinG of KIDS, NA: Not Available, MAS: Massachusetts site, MIN: Minnesota site  *^a^* Fisher’s Exact test for mother’s race and ethnicity; Pearson’s Chi-squared test for all other comparisons  *^b^* Ns and %s for cardiac intervention exclude ATL cases (for whom data was not available)  *^c^* Cells with <10 observations suppressed for known categories | | | | | | | | | |

**Supplemental Table 2. Survey response rates by characteristics at birth and site among children born 2006-2021 with CHD in Atlanta, Georgia, Massachusetts, and Minnesota, CHSTRONG KIDS**

| **Characteristic** | ***ATL*** | | ***MAS*** | | ***MIN*** | |
| --- | --- | --- | --- | --- | --- | --- |
|  | **Respondents n (row %) (N = 491)** | **Non-respondents n (row %) (N = 2100)** | **Respondents n (row %) (N = 510)** | **Non-respondents n (row %) (N = 847)** | **Respondents n (row %) (N = 840)** | **Non-respondents n (row %) (N = 1452)** |
| Sex |  |  |  |  |  |  |
| Male | 233 (18.2%) | 1,044 (81.8%) | 307 (38.1%) | 499 (61.9%) | 461 (38.6%) | 733 (61.4%) |
| Female | 258 (19.7%) | 1,054 (80.3%) | 203 (36.8%) | 348 (63.2%) | 379 (34.5%) | 719 (65.5%) |
| Unknown | 0 | 2 | 0 | 0 | 0 | 0 |
| Year of birth |  |  |  |  |  |  |
| 2006-2011 | 311 (18.0%) | 1,418 (82.0%) | 177 (35.5%) | 321 (64.5%) | 188 (37.8%) | 310 (62.2%) |
| 2012-2017 | 154 (20.4%) | 600 (79.6%) | 207 (39.2%) | 321 (60.8%) | 355 (34.3%) | 680 (65.7%) |
| 2018-2021 | 26 (24.1%) | 82 (75.9%) | 126 (38.1%) | 205 (61.9%) | 297 (39.1%) | 462 (60.9%) |
| Gestational age at birth |  |  |  |  |  |  |
| Preterm (<37 weeks) | 94 (16.5%) | 474 (83.5%) | 75 (37.1%) | 127 (62.9%) | 156 (35.5%) | 284 (64.5%) |
| Full-term (≥37 weeks) | 391 (19.6%) | 1,602 (80.4%) | 434 (37.7%) | 716 (62.3%) | 684 (37.0%) | 1,167 (63.0%) |
| Unknown | 6 | 24 | 1 | 4 | 0 | 1 |
| Birth weight (grams) |  |  |  |  |  |  |
| <2500 (low) | 95 (17.7%) | 441 (82.3%) | 76 (35.5%) | 138 (64.5%) | 146 (32.7%) | 300 (67.3%) |
| ≥2500 (normal) | 392 (19.2%) | 1,646 (80.8%) | 424 (38.1%) | 690 (61.9%) | 694 (37.6%) | 1,152 (62.4%) |
| Unknown | 4 | 13 | 10 | 19 | 0 | 0 |
| Plurality |  |  |  |  |  |  |
| Singleton | 454 (18.8%) | 1,962 (81.2%) | 484 (37.9%) | 794 (62.1%) | 796 (36.7%) | 1,371 (63.3%) |
| Multiple birth | 34 (24.3%) | 106 (75.7%) | 26 (33.3%) | 52 (66.7%) | 44 (35.2%) | 81 (64.8%) |
| Unknown | 3 | 32 | 0 | 1 | 0 | 0 |
| CHD Severity |  |  |  |  |  |  |
| Severe | 220 (23.4%) | 719 (76.6%) | 510 (37.6%) | 847 (62.4%) | 564 (39.2%) | 874 (60.8%) |
| Shunt+Valve | <10*^a^* | 51 (85.0%) | NA | NA | 72 (31.6%) | 156 (68.4%) |
| Shunt | 223 (16.4%) | 1,135 (83.6%) | NA | NA | <10*^a^* | <10*^a^* |
| Valve | 25 (16.9%) | 123 (83.1%) | NA | NA | 200 (32.6%) | 414 (67.4%) |
| Other | 14 (16.3%) | 72 (83.7%) | NA | NA | NA | NA |
| Had a cardiac intervention in the first year of life*^b^* |  |  |  |  |  |  |
| Yes | NA | NA | 366 (38.5%) | 585 (61.5%) | 528 (39.5%) | 808 (60.5%) |
| No | NA | NA | 144 (35.6%) | 261 (64.4%) | 34 (34.3%) | 65 (65.7%) |
| Unknown | NA | NA | 0 | 1 | 278 | 579 |
| Diagnosed with Trisomy 21 |  |  |  |  |  |  |
| Yes | 31 (20.3%) | 122 (79.7%) | 22 (36.7%) | 38 (63.3%) | 100 (42.4%) | 136 (57.6%) |
| No | 460  (18.9%) | 1978 (81.1%) | 488  (37.6%) | 809  (62.4%) | 740  (36.0%) | 1,316  (64.0%) |
| Mother's marital status at delivery |  |  |  |  |  |  |
| Married | 200 (24.2%) | 627 (75.8%) | 362 (43.0%) | 479 (57.0%) | 683 (42.9%) | 910 (57.1%) |
| Unmarried | 59 (10.4%) | 507 (89.6%) | 108 (25.4%) | 318 (74.6%) | 157 (22.5%) | 542 (77.5%) |
| Unknown | 232 | 966 | 40 | 50 | 0 | 0 |
| Mother's age at delivery (years) |  |  |  |  |  |  |
| ≤18 | 12 (13.8%) | 75 (86.2%) | <10*^a^* | 25 (86.2%) | <10*^a^* | 29 (90.6%) |
| 19-24 | 64 (12.4%) | 451 (87.6%) | 43 (21.7%) | 155 (78.3%) | 78 (24.1%) | 246 (75.9%) |
| 25-34 | 255 (19.0%) | 1,088 (81.0%) | 316 (41.4%) | 448 (58.6%) | 549 (38.6%) | 874 (61.4%) |
| ≥35 | 152 (25.3%) | 448 (74.7%) | 147 (40.2%) | 219 (59.8%) | 210 (40.9%) | 303 (59.1%) |
| Unknown | 8 | 38 | 0 | 0 | 0 | 0 |
| Mother's educational attainment at delivery |  |  |  |  |  |  |
| Less than high school | 23 (8.3%) | 254 (91.7%) | 21 (15.9%) | 111 (84.1%) | 28 (12.2%) | 201 (87.8%) |
| High school or equivalent | 58 (10.1%) | 516 (89.9%) | 70 (28.2%) | 178 (71.8%) | 101 (23.4%) | 330 (76.6%) |
| Some college, no degree | 53 (18.0%) | 241 (82.0%) | 62 (31.6%) | 134 (68.4%) | 124 (31.0%) | 276 (69.0%) |
| Associate degree | 25 (23.8%) | 80 (76.2%) | 44 (31.7%) | 95 (68.3%) | 93 (38.1%) | 151 (61.9%) |
| Bachelor's degree | 178 (30.5%) | 406 (69.5%) | 153 (43.1%) | 202 (56.9%) | 294 (48.0%) | 319 (52.0%) |
| Graduate degree | 91 (32.7%) | 187 (67.3%) | 157 (59.2%) | 108 (40.8%) | 193 (55.5%) | 155 (44.5%) |
| Unknown | 63 | 416 | 3 | 19 | 7 | 20 |
| Mother's race and ethnicity |  |  |  |  |  |  |
| Non-Hispanic White | 269 (31.5%) | 586 (68.5%) | 387 (44.8%) | 476 (55.2%) | 713 (43.6%) | 922 (56.4%) |
| Non-Hispanic Black | 140 (15.2%) | 779 (84.8%) | 27 (21.8%) | 97 (78.2%) | 26 (8.9%) | 265 (91.1%) |
| Hispanic | 43 (7.9%) | 499 (92.1%) | 56 (23.9%) | 178 (76.1%) | 43 (25.0%) | 129 (75.0%) |
| Non-Hispanic American Indian and Alaskan Native | 0 (0.0%) | <10*^a^* | 0 (0.0%) | 0 (0.0%) | <10*^a^* | 21 (87.5%) |
| Non-Hispanic Asian or Pacific Islander | 20 (17.4%) | 95 (82.6%) | 33 (34.7%) | 62 (65.3%) | 46 (31.1%) | 102 (68.9%) |
| Non-Hispanic Other | 0 (0.0%) | <10*^a^* | <10*^a^* | 16 (94.1%) | <10*^a^* | <10*^a^* |
| Unknown | 19 | 133 | 6 | 18 | 6 | 10 |
| Rurality of birth residence |  |  |  |  |  |  |
| Metro | 491  (19.0%) | 2,096  (81.0%) | 506  (37.7%) | 835  (62.3%) | 727  (36.7%) | 1,253  (63.3%) |
| Non-metro | 0 | 0 | <10*^a^* | 11  (73.3%) | 113  (36.2%) | 199  (63.8%) |
| Unknown | 0 | 4 | 0 | 1 | 0 | 0 |

ATL: Atlanta site, CHD: Congenital heart defects, CHSTRONG KIDS: Congenital Heart Survey To Recognize Outcomes, Needs, and well-beinG of KIDS, MAS: Massachusetts site, MIN: Minnesota site

*^a^* Cells with <10 observations suppressed for known categories

*^b^* Ns and %s for cardiac intervention exclude ATL cases (for whom data was not available)

**Supplemental Table 3. Survey response rates by characteristics at birth among 3,103 children eligible for CHSTRONG KIDS – limited to heart defect diagnoses collected by all 3 sites**

| **Characteristic** | **Respondents n (row %)** (N = 1096) | **Non-respondents n (row %)** (N = 2007) | **p-value***^a^* | |
| --- | --- | --- | --- | --- |
| Site |  |  | <0.001 | |
| ATL | 177 (26.1%) | 500 (73.9%) |  | |
| MAS | 506 (37.6%) | 838 (62.4%) |  | |
| MIN | 413 (38.2%) | 669 (61.8%) |  | |
| Sex |  |  | 0.59 | |
| Male | 658 (35.7%) | 1,185 (64.3%) |  | |
| Female | 438 (34.8%) | 822 (65.2%) |  | |
| Unknown | 0 | 0 |  | |
| Year of birth |  |  | 0.18 | |
| 2006-2011 | 378 (33.7%) | 745 (66.3%) |  | |
| 2012-2017 | 426 (35.3%) | 782 (64.7%) |  | |
| 2018-2021 | 292 (37.8%) | 480 (62.2%) |  | |
| Gestational age at birth |  |  | 0.34 | |
| Preterm (<37 weeks) | 162 (33.5%) | 322 (66.5%) |  | |
| Full-term (≥ 37 weeks) | 929 (35.7%) | 1,670 (64.3%) |  | |
| Unknown | 5 | 15 |  | |
| Birth weight (grams) |  |  | 0.33 | |
| <2500 (low) | 177 (33.5%) | 351 (66.5%) |  | |
| ≥2500 (normal) | 906 (35.8%) | 1,628 (64.2%) |  | |
| Unknown | 13 | 28 |  | |
| Plurality |  |  | 0.49 | |
| Singleton | 1,031 (35.4%) | 1,883 (64.6%) |  | |
| Multiple birth | 62 (38.0%) | 101 (62.0%) |  | |
| Unknown | 3 | 23 |  | |
| CHD Severity |  |  |  | |
| Severe | 1,096 (35.3%) | 2,007 (64.7%) |  | |
| Shunt+Valve | 0 | 0 |  | |
| Shunt | 0 | 0 |  | |
| Valve | 0 | 0 |  | |
| Other | 0 | 0 |  | |
| Had a cardiac intervention in the first year of life*^b^* |  |  | 0.31 | |
| Yes | 724 (38.4%) | 1,159 (61.6%) |  | |
| No | 147 (35.8%) | 264 (64.2%) |  | |
| Unknown | 225 | 584 |  |  |
| Has Trisomy 21 |  |  | 0.80 |  |
| Yes | 39 (36.4%) | 68 (63.6%) |  | |
| No | 1,057  (35.3%) | 1,939  (64.7%) |  | |
| Mother's marital status at delivery |  |  | <0.001 | |
| Married | 757 (42.0%) | 1,046 (58.0%) |  | |
| Unmarried | 212 (23.7%) | 683 (76.3%) |  | |
| Unknown | 127 | 278 |  | |
| Mother's age at delivery |  |  | <0.001 | |
| ≤18 | <10*^c^* | 56 (86.2%) |  | |
| 19-24 | 107 (22.3%) | 373 (77.7%) |  | |
| 25-34 | 688 (37.7%) | 1,139 (62.3%) |  | |
| ≥35 | 291 (40.2%) | 433 (59.8%) |  | |
| Unknown | 1 | 6 |  | |
| Mother's educational attainment at delivery |  |  | <0.001 | |
| Less than high school | 39 (12.8%) | 266 (87.2%) |  | |
| High school diploma or equivalent | 140 (22.9%) | 472 (77.1%) |  | |
| Some college, no degree | 152 (32.4%) | 317 (67.6%) |  | |
| Associate degree | 96 (35.0%) | 178 (65.0%) |  | |
| Bachelor's degree | 356 (44.7%) | 441 (55.3%) |  | |
| Graduate degree | 290 (56.6%) | 222 (43.4%) |  | |
| Unknown | 23 | 111 |  | |
| Mother's race and ethnicity |  |  | <0.001 | |
| Non-Hispanic White | 842 (44.6%) | 1,047 (55.4%) |  | |
| Non-Hispanic Black | 93 (18.8%) | 403 (81.3%) |  | |
| Hispanic | 83 (20.3%) | 326 (79.7%) |  | |
| Non-Hispanic American Indian and Alaskan Native | <10*^c^* | 11 (78.6%) |  | |
| Non-Hispanic Asian or Pacific Islander | 59 (27.2%) | 158 (72.8%) |  | |
| Non-Hispanic Other | <10*^c^* | 23  (88.5%) |  | |
| Unknown | 13 | 39 |  | |
| Rurality of birth residence |  |  | 0.68 | |
| Metro | 1,037  (35.4%) | 1,890  (64.6%) |  | |
| Non-metro | 59  (33.9%) | 115  (66.1%) |  | |
| Unknown | 0 | 2 |  | |

ATL: Atlanta site, CHD: Congenital heart defects, CHSTRONG KIDS: Congenital Heart Survey To Recognize Outcomes, Needs, and well-beinG of KIDS, MAS: Massachusetts site, MIN: Minnesota site

*^a^* Fisher’s Exact test for mother's race and ethnicity; Pearson’s Chi-squared test for all other comparisons

*^b^* Ns and %s for cardiac intervention exclude ATL cases (for whom data was not available)

*^c^* Cells with <10 observations suppressed for known categories

**Supplemental Figure 2. Percentage of children with select types of severe CHD who were not up-to-date on their cardiology care*^a^*, CHSTRONG KIDS – limited to heart defect diagnoses collected by all 3 sites**

CHD: Congenital heart defects, CHSTRONG KIDS: Congenital Heart Survey To Recognize Outcomes, Needs, and well-beinG of KIDS, ATL= Atlanta, Georgia, MAS = Massachusetts, MIN: Minnesota

*^a^* According to recommendations from the 2018 American Heart Association/American College of Cardiology Guideline for the Management of Adults with Congenital Heart Disease [5]

*^b^* Post-stratification weights were calculated for each survey using data on site, child sex, maternal race and ethnicity, child’s age, and CHD severity from the birth defect surveillance system
